# Adipocyte‐specific FFA2 deletion leads to increased adipose inflammation and is associated with altered intestinal lipid handling in mice

**DOI:** 10.14814/phy2.70875

**Published:** 2026-05-04

**Authors:** Chioma Nnyamah, James Boyett, Barton Wicksteed, Nupur Pandya, Kai Xu, Irene Corona‐Avila, Nadia Sweis, Marissa St. George, Laura J. Den Hartigh, Abeer M. Mahmoud, Yuwei Jiang, Jose Cordoba‐Chacon, Medha Priyadarshini, Brian T. Layden

**Affiliations:** ^1^ Division of Endocrinology, Diabetes and Metabolism, Department of Medicine University of Illinois Chicago Chicago Illinois USA; ^2^ Division of Metabolism, Endocrinology and Nutrition University of Washington Seattle Washington USA; ^3^ Jesse Brown Veterans Affairs Medical Center Chicago Illinois USA

**Keywords:** adipose tissue, dietary fiber, free fatty acid receptor 2, gut‐adipose axis, metabolic dysfunction, short chain fatty acids

## Abstract

Obesity and related metabolic disorders are often characterized by chronic adipose tissue inflammation, driving systemic insulin resistance and general metabolic dysfunction. Free Fatty Acid Receptor 2 (FFA2) has emerged as a potential modulator of adipocyte function, inflammation, and metabolism. To investigate the role of FFA2 expressed in the adipose tissue, we generated adipose‐specific FFA2 knockout mice (Adipoq‐F2‐KO) and assessed metabolic outcomes under standard laboratory chow and high‐fat, high‐sugar Western diet conditions, with and without dietary fiber supplementation. We found that adipose‐specific FFA2 deletion had minimal metabolic consequences under standard dietary conditions but significantly reduced body weight and adiposity when mice were fed a fiber (fructooligosaccharide)‐supplemented Western diet. Subsequent fecal analyses and transcriptomic profiling indicated impaired intestinal lipid absorption as the primary driver of reduced adiposity, suggesting disrupted adipose‐intestinal communication. Unexpectedly, the lighter Adipoq‐F2‐KO mice also exhibited heightened adipose inflammation, characterized by increased macrophage infiltration and pro‐inflammatory cytokine expression. Furthermore, in vitro loss‐of‐function experiments in adipocytes revealed that FFA2 knockdown impaired adipocyte maturation, lipid storage, and anti‐inflammatory signaling. Additional studies using intestinal epithelial cells exposed to adipocyte‐conditioned media implicated adipose‐derived signals in driving intestinal dysfunction. Collectively, our findings highlight adipose‐specific FFA2 as critical in regulating adipose tissue inflammation, lipid metabolism, and inter‐organ communication.

## 1 INTRODUCTION

Obesity and associated metabolic disorders, such as insulin resistance, type 2 diabetes, and cardiovascular disease, are critical public health issues worldwide. These conditions are largely associated with chronic, low‐grade inflammation that partly stems from dysfunction in adipose tissue (de Heredia et al., 2012; Hotamisligil, 2006). As adipocytes expand beyond vascular growth during obesity, they become hypoxic, producing pro‐inflammatory cytokines like TNF‐α and MCP‐1, which promote macrophage recruitment. This cycle of inflammation worsens insulin resistance and metabolic dysfunction. Emerging evidence shows that this persistent inflammation is a major contributor to the development of metabolic syndrome (Kahn et al., 2006; Lumeng & Saltiel, 2011).

Of importance, adipose tissue serves as both an energy storage organ and an endocrine organ that influences systemic metabolism through hormone (adipokine) secretion. Thus, understanding how adipose tissue regulates energy balance and metabolic health is crucial. Approaches to modulate adipose tissue activity, including pharmacological approaches targeting adipocyte signaling pathways (Hausman et al., 2001), the promotion of browning in white adipocytes (Kuryłowicz & Puzianowska‐Kuźnicka, 2020), and dietary interventions to enhance fat metabolism (Chao et al., 2021), are being explored. Interestingly, obesity has been associated with changes in the gut microbiota (GM), possibly via microbial metabolites that influence host physiology (Hur & Lee, 2015). Microbial metabolites such as short‐chain fatty acids (SCFAs), indoles, tryptophan derivatives, and branched‐chain amino acids have been shown to regulate immune function, metabolic signaling, and inflammation (Li et al., 2021; Seo & Kwon, 2023; Shen et al., 2024). These GM metabolites can act locally in the gut or systemically, where they influence metabolic outcomes by interacting with various nutrient‐sensing and energy‐regulating organs, including the brain, liver, pancreas, skeletal muscle, and adipose tissue (Liu, Tan, et al., 2022). In adipose tissue, these metabolites have been suggested to impact adipogenesis, lipid storage, lipolysis, insulin signaling, extracellular matrix remodeling, and inflammation (Liu, Yang, et al., 2022).

Some of these adipose‐specific effects have been reported to be modulated by Free Fatty Acid Receptor 2 (FFA2/GPR43) (al Mahri et al., 2022; Kimura et al., 2020). FFA2 is primarily activated by acetate, with a lower affinity for propionate and butyrate, and is highly expressed in adipose tissue, the pancreas, the immune system, and the gut (NCBI, n.d.). However, results from studies aimed at uncovering the role of FFA2 in metabolism have been discordant. Priyadarshini et al. reported that FFA2 knockout (KO) mice gained less weight and had improved insulin sensitivity under high‐fat diet conditions (Priyadarshini et al., 2015), contrasting results from Kimura et al. (2013). A possible explanation for these conflicting results is the use of global KO models where FFA2 is deleted from all the tissues, as FFA2 may exert tissue‐specific effects with different roles in adipose tissue compared to other organs. Additionally, most studies on FFA2 have been conducted without dietary supplementation of fermentable fibers, the dietary precursors to SCFAs that activate the receptor. As such, the role of FFA2 in metabolic regulation may be underestimated or misinterpreted without sufficient ligand levels and/or activation.

To address these gaps, we developed an adipose‐specific FFA2 KO model by crossing Adiponectin‐Cre mice with floxed FFA2 mice (Adipoq‐F2‐KO). After observing that Adipoq‐F2‐KO mice were metabolically comparable to their control (floxed) counterparts on a standard laboratory chow (SC) diet, we subjected them to a high‐fat, high‐sugar Western diet (WD), with and without fructooligosaccharides (FOS), to provide the GM with fermentable substrates necessary for SCFA production relevant to the receptor of interest. We observed that Adipoq‐F2‐KO mice were consistently lighter than controls when fed a fiber‐supplemented WD despite showing no significant changes in glucose homeostasis, food intake, or energy expenditure (EE). Surprisingly, these lighter Adipoq‐F2‐KO mice exhibited persistently heightened adipose inflammation, suggesting that FFA2 deletion in adipocytes may inherently drive a pro‐inflammatory state. In vitro studies using FFA2 knockdown 3T3L1 adipocytes also showed that FFA2 is required for proper adipocyte maturation, lipid storage, and the suppression of pro‐inflammatory signaling. Additionally, transcriptomic and fecal lipid analyses implicate disrupted lipid absorption as a key mechanism underlying the reduced weight gain of the Adipoq‐F2‐KO mice and point to adipose‐intestinal crosstalk. Future studies in our lab will aim to delineate how FFA2 orchestrates signals between adipose tissue and the gut, with the ultimate goal of leveraging this receptor as a potential target for therapeutic intervention in obesity‐associated metabolic diseases.

## 2 MATERIALS AND METHODS

### 2.1 Animal models

All animal experiments were approved by the Institutional Animal Care and Use Committee (IACUC) at the University of Illinois at Chicago (UIC). Adipoq‐F2‐KO experimental mice were generated by crossing FFA2 floxed control mice (*ffa2* ^*fl/fl*^ initially described in (Lednovich et al., 2024)) with Adiponectin‐Cre (Adipoq‐Cre, RRID: IMSR_JAX:028020, Jackson Laboratory) mice. Experimental mice of both genotypes were co‐housed from birth to 12 weeks of age under a temperature‐controlled environment with a 12‐h light/dark cycle and full access to a standard laboratory chow (Teklad LM‐485, Envigo, Indianapolis, IN, USA) and water. Some cohorts transitioned to varying temperatures and/or custom‐modified Western diet (WD) (#D19010907 with corn‐starch replaced by Amioca) or WD supplemented with 10% Fructooligosaccharides (FOS, #D20021208, oligosaccharide chain length of 2–10 units) obtained from Research Diets, Inc., New Brunswick, NJ, USA, as detailed in the results section. Animals were monitored for body weight at specified time points. Mice were selected for inclusion based on successful genotyping and absence of gross developmental abnormalities. Animals were excluded from analysis if they exhibited illness, failure to thrive, or did not complete the assigned dietary intervention, as noted next. A total of 11 animals were excluded across all cohorts as these mice did not complete their dietary intervention for the following reasons: 3 for illness (dermatitis requiring veterinary intervention), 4 for failure to thrive (body weight >2 SD below cohort mean at weaning), and 4 for incomplete dietary intervention (2 due to cage flooding requiring early euthanasia, 2 due to fighting requiring separation).

Both male and female mice were initially included in the study and underwent identical metabolic phenotyping procedures. However, female mice of both genotypes did not display a significant weight gain response to the Western diet. As the study objective was to characterize obesity‐associated phenotypes, subsequent cohorts and analyses were conducted exclusively in males. Experimental animals were randomly assigned to dietary or temperature exposure groups within each genotype to reduce allocation bias. Sample sizes were determined based on power studies from prior studies using similar models and endpoints (Lednovich et al., 2022), as well as feasibility considerations. Animal cohorts consisted of *n* = 5–7 mice per genotype group for each dietary intervention. Specific group sizes for individual experiments are provided in the figure legends and ranged from *n* = 3–8 per group depending on the assay. Formal power calculations were not performed.

Investigators were blinded to genotype during data collection for key metabolic phenotyping, histological analyses, and image quantification to minimize observer bias. At the end of the 24‐week period, mice were euthanized under isoflurane anesthesia, and their tissues were immediately dissected, weighed (reported relative to total body weight), and frozen in liquid nitrogen and stored at −80°C until use.

### 2.2 Body composition measurements

For thermoneutral and cold cohorts, body composition was measured instead of individual depot weights to provide a more holistic assessment of adiposity. Body composition measurements were collected via Nuclear Magnetic Resonance (NMR) with the Bruker Minispec LF50 Body Composition Analyzer (Bruker Corporation, Billerica, MA, USA).

### 2.3 Glucose homeostasis measurements

Intra‐peritoneal glucose tolerance tests (IPGTTs), Intra‐peritoneal insulin tolerance tests (IPITTs), and fasting glucose/insulin measurements were performed as previously reported (Lednovich et al., 2022; Lednovich et al., 2024).

### 2.4 Non‐esterified fatty acid (NEFA), cholesterol, and triglyceride measurements

NEFA, cholesterol, and triglyceride were quantified in plasma and feces using the Wako HR series NEFA‐HR (2) enzymatic colorimetric method assay (cat# 999‐34,691/995‐34,791/991‐34,891/993‐35,191), Wako Cholesterol E enzymatic colorimetric assay (cat# 439‐17,501), and the Wako L‐Type TG M test (cat# 461‐08992/457‐09092), respectively. Fecal lipid extraction and quantification were conducted according to the protocol by Kraus et al. (Kraus et al., 2015), with some modifications: post‐drying, samples were re‐dissolved in 1 mL ddH_2_O (instead of being weighed) and then assayed for NEFA levels.

### 2.5 Food intake measurements

Mice were individually housed 6 weeks after the WD + FOS diet challenge in a BioDaq food intake monitoring system (Research Diets Inc.). They were acclimated to the system for 72 h before being transferred to active reading cages for a 96‐h monitoring period.

### 2.6 Metabolic cages and indirect calorimetry

Indirect calorimetry was performed as previously reported (Lednovich et al., 2022). For thermoneutrality assessments, the temperature was gradually increased to 30°C over the first 6 h of data recording. Data were processed and analyzed using the CalR Metabolic Cage Analysis platform (CalRapp.org), applying the “Two Groups” option for statistical comparisons. Energy expenditure analysis was performed using ANCOVA, with total body mass, fat mass, or lean mass as a covariate.

### 2.7 RNA extraction and qPCR

These studies were performed as reported in our previous work (Lednovich et al., 2022) using TRIzol reagent (Life Technologies) with chloroform for phase separation for tissues and RLT lysis buffer (cat# 79216, Qiagen) for cultured cells. Purification was performed with the RNeasy Mini Kit (cat# 74106, Qiagen), and samples were treated with RNase‐free DNase (cat# 79254, Qiagen) to remove residual genomic DNA. 1 μg of purified RNA was reverse transcribed using iScript Reverse Transcription Kit (cat# 1708891, Bio‐Rad Laboratories, Hercules, CA) and amplified using SYBR Green SuperMix (cat# 1725124, Bio‐Rad). FFA2 knockout and knockdown efficiency were confirmed at the mRNA level by qPCR, as commercially available FFA2/FFAR2 antibodies lack sufficient specificity and reliability for protein‐level validation in mouse adipose tissue and 3 T3‐L1 cells.

### 2.8 RNASeq

RNA sequencing was conducted using jejunum and mature white adipocyte samples isolated from epididymal white adipose tissue (eWAT), with services provided by NUSeq Core Facility (Northwestern University, Chicago, IL) for jejunum samples and the UIC sequencing core for adipocyte samples. RNA integrity was confirmed using the Agilent Bioanalyzer, and library construction and sequencing were carried out using an Illumina NovaSeq X Plus platform. Raw reads were trimmed using Trimmomatic with standard Illumina TruSeq adapter sequences. Trimmed reads were aligned to the mouse reference genome using the STAR aligner, and gene‐level counts were generated using featureCounts. Differential expression analysis and normalization were performed using DESeq2.

The adipocyte RNASeq dataset and jejunum RNASeq dataset have been deposited in the NCBI Gene Expression Omnibus (GEO) under accession numbers GSE302985 and GSE309021, respectively.

### 2.9 Pathway analysis

All pathway analyses were performed utilizing differential expression analysis from bulk RNAseq, with significance cut‐off points for differential expression between genotypes as FDR <0.05 and *p*‐value <0.05 for adipocyte and jejunal RNAseq, respectively. Significantly upregulated genes per genotype were then processed with DAVID (RRID:SCR_001881) (Huang et al., 2009; Sherman et al., 2022) versus the *Mus musculus* annotated gene list, followed by KEGG (RRID:SCR_012773) and Reactome pathway analysis. Results from pathway analysis were then analyzed via R to select significantly upregulated pathways per genotype based on *p*‐value <0.05 and plotted based on negative *p*‐value.

### 2.10 Histology and immunofluorescence staining

Sample preservation and sectioning were done according to the methods outlined in our previous work (Lednovich et al., 2022). F4/80 was visualized using anti‐F4/80 antibody (Abcam ab11101, Cambridge, UK) with Goat Anti‐Rat Alexafluor 594 antibody (Invitrogen – A11007, Carlsbad, CA). Coverslips were mounted with Invitrogen Prolong Gold Antifade Reagent with DAPI (cat# P36931, Invitrogen), and slides were viewed with a Leica DMi8 microscope (Leica Biosystems, Wetzlar, Germany). Fluorescence was quantified using ImageJ (RRID:SCR_003070) after background and noise subtraction.

### 2.11 3T3L1 F2‐KD cell line generation and use

3 T3‐L1 preadipocytes were obtained from ATCC (CL‐173, ATCC, Manassas, VA, RRID: CVCL_0123) and expanded in culture following the ATCC‐recommended handling instructions (American Type Culture Collection, n.d.). For gene knockdown (KD) experiments, cells were infected with a custom‐designed lentivirus carrying either an empty vector (EV) or a shRNA specific for FFA2 (VectorBuilder Inc., Chicago, IL, vector IDs: VB221122‐1200stq, VB010000‐9298rtf). Lentiviral transduction was performed using 6 μg/mL polybrene (cat# TR‐1003‐G, Millipore‐Sigma) to enhance infection efficiency. Cells were incubated with the viral particles for 24 h, after which the medium was replaced with a fresh growth medium. GFP expression was used to confirm successful transduction, and knockdown efficiency was assessed via qPCR and the generation of EV control 3T3L1s and FFA2 KD (F2KD) 3T3L1s.

Cell lines were routinely monitored for expected morphology (spindle‐like appearance pre‐differentiation and internal lipid “bubbles” post‐differentiation) and preadipocyte/adipocyte marker expression (pref1, adiponectin) and were screened for mycoplasma contamination every 4–6 weeks using a PCR‐based detection kit (Lonza MycoAlert, catalog #LT07‐318). No contamination was detected throughout the study. After the knockdown was established and confirmed to be stable across multiple passages, 3 T3‐L1 EV and F2KD preadipocytes were differentiated following a standard two‐phase differentiation protocol (doi: 10.1016/j.ab.2012.03.005.) (American Type Culture Collection, n.d.). For acetate treatments, sodium acetate stock solutions were prepared at 100 mM in differentiation media, sterile‐filtered, and diluted to the experimental concentration prior to treatment. Fully differentiated 3T3L1 adipocyte‐like cells were treated with acetate‐supplemented media for the designated experimental period before further analysis.

### 2.12 IEC18 transwell culture

IEC18 rat intestinal epithelial cells were selected for these proof‐of‐concept experiments as they are a well‐established model for studying epithelial responses to secreted factors in transwell systems. While mouse‐origin intestinal cells would provide better species matching, the conserved nature of mammalian intestinal signaling pathways and the focus on soluble mediators (rather than species‐specific cell–cell interactions) support the validity of this approach for testing whether adipocyte‐derived factors can influence intestinal epithelial gene expression. These cells were cultured until they reached confluence on Corning Transwell 12‐well plate inserts with 0.4 μm pore size polycarbonate membranes (CLS3422, Millipore‐Sigma, Burlington, MA). Once confluent, IEC18 cells were exposed to conditioned media (CM) from differentiated 3 T3‐L1s, as described in the *Results* section. At the end of the treatment period, transwell inserts were carefully removed, the membranes were gently rinsed in cold PBS, and cells were scraped in lysis buffer. RNA was isolated as described above.

### 2.13 Western blot analysis

Western blotting was done according to the BioRad standard protocol for Western Blotting (no doi available) (Bio‐Rad Laboratories, Inc., 2017) with primary antibodies against pERK1/2 (cat# 4370, Cell Signaling Technology), total ERK1/2 (cat# 4695, Cell Signaling Technology), or GAPDH (cat# 5174, Cell Signaling Technology) as the loading control. After washing, membranes were incubated with HRP‐conjugated secondary antibodies (cat# 7074, Cell Signaling Technology) and developed using an ECL detection system (cat# 1705061, Bio‐Rad). Bands were visualized using the Chemidoc MP Imaging System (Biorad, SCR_021693).

### 2.14 Oil red O staining

Oil red O staining of 3 T3‐L1 cells was done according to the protocol published by Kraus et al. (doi: 10.1080/21623945.2016.1240137) using oil red powdered reagent (cat# O0625, Sigma‐Aldrich) (Kraus et al., 2016).

### 2.15 Statistical analyses

All data are reported as mean ± standard error of the mean (SEM). *Justification*: SEM is the appropriate measure of variability for this study as our primary objective is to make inferences about population‐level differences between genotypes through hypothesis testing (*t*‐tests, ANOVA) rather than to characterize within‐group dispersion. SEM reflects the precision of the sample mean as an estimate of the true population mean and is consistent with the statistical framework employed throughout this manuscript. This approach aligns with established conventions in metabolic physiology research and our previously published work (Lednovich et al., 2022).

Statistical analyses were performed using GraphPad Prism version 10.0 (RRID:SCR_002798). Prior to hypothesis testing, data distributions were assessed for normality using the Shapiro–Wilk test, and equality of variances was evaluated using Levene's test. For comparisons between two groups, two‐tailed unpaired Student's *t*‐tests were used, assuming equal variances and independent sampling. When comparing more than two groups, either one‐way or two‐way analysis of variance (ANOVA) was applied depending on the number of independent variables. One‐way ANOVA was used for single‐factor comparisons (e.g., genotype) and followed by Tukey's post hoc test for multiple comparisons. Two‐way ANOVA was used when evaluating interactions between genotype and either treatment or time, and post hoc significance was determined using Sidak's multiple comparisons test. When analyzing energy expenditure, analysis of covariance (ANCOVA) was applied, with total body mass, lean mass, or fat mass included as covariates as appropriate. ANCOVA assumptions, including homogeneity of regression slopes and linearity, were verified prior to interpretation. A threshold of *p* < 0.05 was used to determine statistical significance. Sample sizes, test statistics, and exact *p*‐values are reported in the figure panels and/or figure legends.

## 3 RESULTS

### 3.1 Adipose‐specific knockout of FFA2 does not Alter metabolic phenotype on standard laboratory chow

**FIGURE 1:**
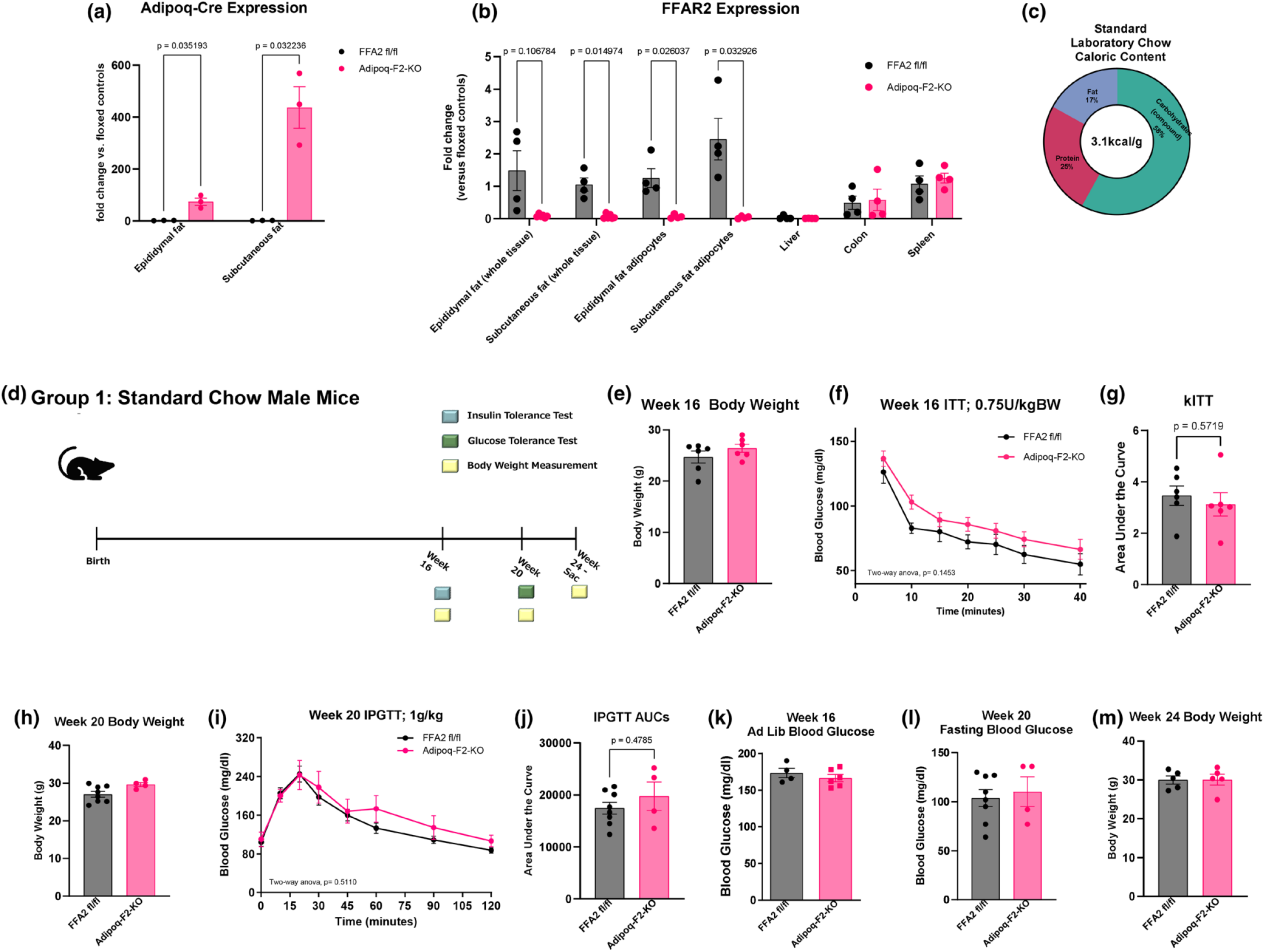
Adipose‐specific knockout of FFA2 does not alter basic metabolic phenotype under standard laboratory chow conditions. (a) Adiponectin‐Cre transgene expression. qPCR using primers targeting the junction between the adiponectin promoter and Cre‐recombinase coding sequence confirms that the Adiponectin‐Cre transgene is highly expressed in adipose tissue, driving specific deletion of FFA2 in mature white adipocytes, *n* = 3 per group. (b) Validation of FFA2 knockout efficiency. FFA2 mRNA was significantly reduced in epididymal and subcutaneous white adipose depots of Adipoq‐F2‐KO mice, with no changes detected in non‐adipose tissues (liver, colon, spleen), *n* = 4 per group. (c) Composition of the standard laboratory chow (SC) diet, emphasizing its low‐fat, low‐sugar content. (d) Experimental timeline– mice were fed the SC diet from weaning through 24 weeks of age for baseline phenotyping. (e, h, m) Body weights of Adipoq‐F2‐KO vs. floxed controls were measured at regular intervals; no differences were observed, *n* = 4–6 per group. (f, g) Intraperitoneal insulin tolerance test (IPITT) showed similar insulin sensitivity between genotypes, *n* = 6 per group. (i, j) Intraperitoneal glucose tolerance test (IPGTT) revealed comparable glucose clearance, *n* = 4–7 per group. (k, l) Ad libitum versus fasting blood glucose measurements were also unchanged, *n* = 4–7 per group. Data are presented as mean ± SEM; statistical significance was assessed by two‐way ANOVA (for time courses) or Student's *t*‐tests (for single time points), with *p* < 0.05 considered significant.

To explore the role of adipose‐specific FFA2 in metabolic regulation, we generated Adipoq‐F2‐KO mice and verified that the Adipoq‐Cre transgene was robustly expressed in the adipose depots (Figure 1a). As anticipated, in Adipoq‐F2‐KO mice, *FFA2* mRNA levels were significantly reduced in adipose tissue, particularly in mature white adipocytes isolated from fractionated, fresh adipose tissue, while remaining unaffected in non‐adipose tissues, such as the liver, spleen, and colon (Figure 1b). To establish a baseline for metabolic challenge and ensure the absence of congenital defects, male and female Adipoq‐F2‐KO and control mice fed SC diet (where the fiber content was primarily composed of insoluble/non‐fermentable fiber) (Figure 1c) were periodically weighed and subjected to basic metabolic testing over 24 weeks (Figure 1d). No significant differences were observed in body weight (Figure 1e,h,m and Figure S1), ad libitum or fasting blood glucose levels (Figure 1k,l) between Adipoq‐F2‐KO and control mice. Next, we tested the overall glucose homeostasis using intraperitoneal insulin tolerance tests (IPITT) and intraperitoneal glucose tolerance tests (IPGTT). During the IPITT, Adipoq‐F2‐KO and control mice showed similar reduced plasma glucose levels in response to injected insulin (Figure 1f,g). Mice also exhibited comparable glucose clearance in response to elevated plasma glucose during the IPGTT (Figure 1i,j). These data demonstrate that under an SC diet, adipose‐specific deletion of FFA2 does not result in detectable metabolic differences between Adipoq‐F2‐KO and control mice. These data indicate that FFA2 in adipose tissue may not significantly regulate glucose metabolism or body weight under physiological conditions.

### 3.2 Adipose‐specific knockout of FFA2 does not significantly impact metabolic responses to a high‐fat, high‐sugar western diet

**FIGURE 2:**
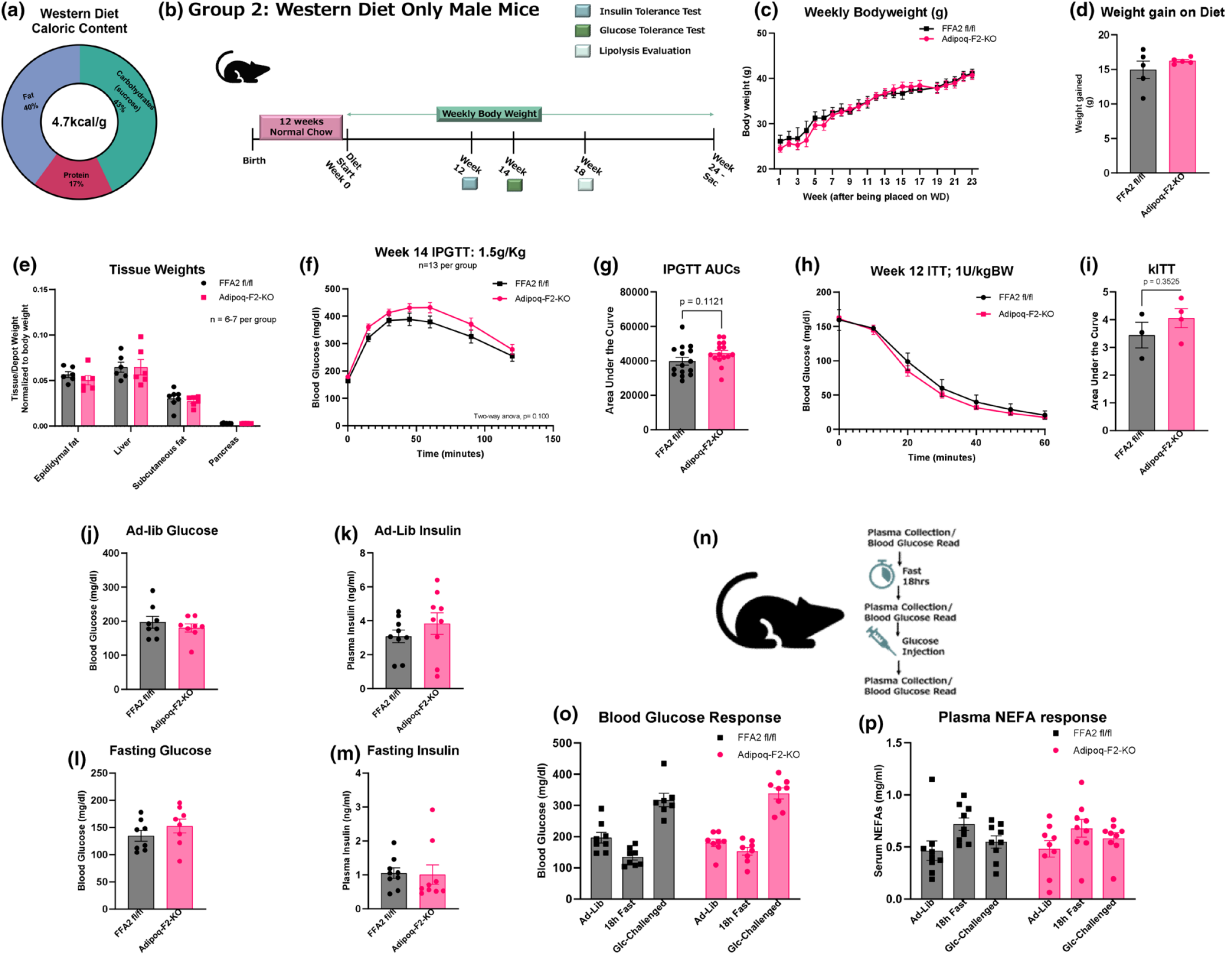
Adipose‐specific FFA2 deletion does not significantly affect metabolic outcomes under a Western diet (WD) lacking fermentable fiber. (a) Western diet composition, highlighting high fat and high sugar (sucrose). (b) Study design: After 12 weeks on normal chow, mice were transitioned to the WD for 24 weeks. (c, d) Body weight gains in male mice show no genotype‐dependent differences throughout WD feeding, *n* = 5 per group. (e) Tissue weights show no genotype‐specific differences, *n* = 5–6 per group. (f, g) IPGTT performed at week 14 on WD showed no differences in glucose clearance in Adipoq‐F2‐KO mice, confirmed by area under the curve (AUC), *n* = 11–13 per group. (h, i) IPITT indicated similar insulin sensitivity between the two genotypes. Data represent a pilot subset (*n* = 3), with full cohort data presented in (a–h). (j–m) Blood glucose (ad libitum and fasting) and insulin measurements were comparable in Adipoq‐F2‐KO vs. floxed controls, *n* = 8 per group. (n–p) Fasting‐induced lipolysis was assessed by serum NEFA concentrations in ad libitum, 18 h‐fasted, and glucose‐challenged states; no differences were detected, *n* = 7–8 per group. Data are shown as mean ± SEM; statistical analysis by two‐way ANOVA for time courses and Student's *t*‐tests for single comparisons.

To assess the role of adipose‐specific FFA2 in regulating metabolic outcomes under obesogenic conditions, we next placed male and female Adipoq‐F2‐KO mice and their littermate controls on a WD after a 12‐week maturation period. The carbohydrate content of this diet was primarily derived from sucrose, providing a significant metabolic challenge for the mice over the course of 24 weeks (Figure 2a and Figure S2). Monitoring various metabolic parameters over the 24‐week study (Figure 2b), we observed that Adipoq‐F2‐KO and control mice gained weight at similar rates (Figure 2c–e). For the female mice, both genotypes were protected from WD‐induced weight gain and metabolic dysfunction (Figure S2), suggesting, as reported before, that the presence of female sex hormones is more critical than FFA2 in regulating metabolic function. (Huang et al., 2020). These findings prompted us to focus our studies on male mice.

Given the reported role of FFA2 in adipocyte function and metabolic regulation (Bolognini et al., 2021), we next examined glucose homeostasis in both genotypes. After 14 weeks of dietary treatment, Adipoq‐F2‐KO mice showed a slight impairment in glucose clearance compared to controls during the IPGTT; however, this trend did not approach statistical significance (Figure 2f,g). Similarly, no differences between the genotypes were noted in insulin tolerance (Figure 2h,i), ad‐libitum and fasting blood glucose and insulin levels (Figure 2j–m). These findings reinforce the conclusion that the absence of FFA2 in adipose tissue does not cause overt disturbances in glucose homeostasis under a WD challenge.

Next, given the role of adipose tissue in lipid metabolism (Ge et al., 2008), we investigated whether adipose‐specific FFA2 deletion might impact lipid handling. To do this, we performed a fasting‐induced lipolysis experiment, measuring serum NEFA levels in ad‐lib, 18‐h‐fasted, and fed states, which allowed us to assess, first, the impact of FFA2 on the ability of adipose tissue to undergo catecholamine‐stimulated lipolysis during fasting or caloric restriction, and second, its capacity to respond to glucose‐stimulated insulin secretion via suppression of lipolysis. Our results showed that NEFA release in response to these challenges was comparable between Adipoq‐F2‐KO mice and controls, suggesting that, under these conditions, adipose‐specific FFA2 KO does not significantly impair fasting‐induced lipolysis or the response to endogenous insulin secretion (Figure 2n–p).

Plasma insulin levels measured during this glucose challenge (data not shown) confirmed comparable insulin secretion between genotypes, consistent with a preserved glucose‐stimulated insulin response. However, future experiments will sample at 5 and 10 min to more closely match the reported insulin secretion windows. Additional experiments using isoproterenol to assess β‐adrenergic‐stimulated lipolysis also revealed no genotype differences between Adipoq‐F2‐KO and control mice (data not shown). Overall, these findings suggest that in the absence of FFA2, the role of adipose tissue in modulating weight gain, glucose handling, and lipid metabolism in response to WD is not significantly impaired, and the deletion of FFA2 has a negligible impact on metabolic outcomes in response to WD.

### 3.3 Adipoq‐F2‐KO mice are significantly lighter than Floxed controls when fed Western diet supplemented with 10% fermentable fiber

**FIGURE 3:**
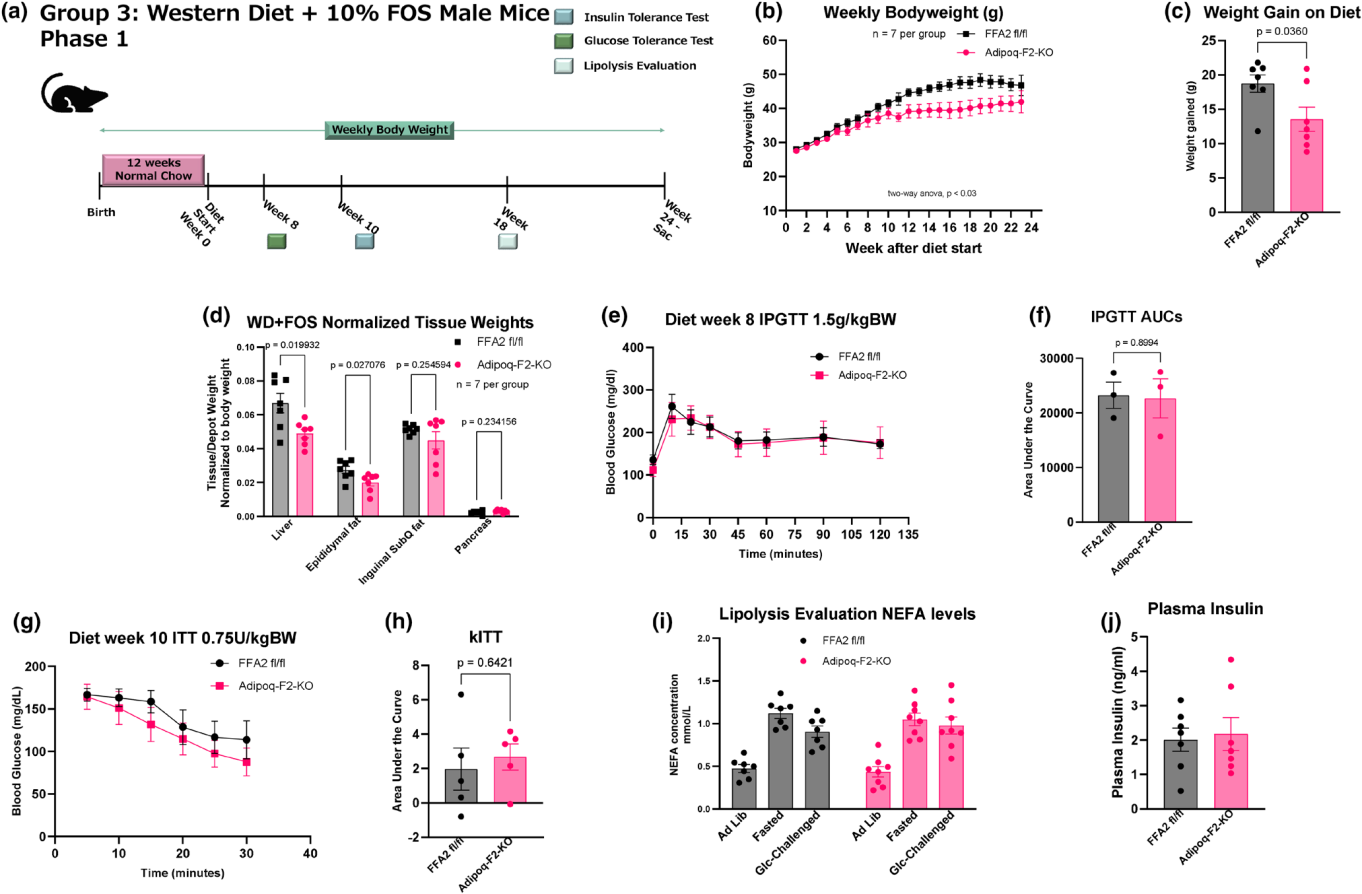
Adipoq‐F2‐KO mice gain significantly less weight than floxed controls on Western diet supplemented with 10% fermentable fiber (FOS). (a) Study design: After 12 weeks on normal chow, mice were transitioned to a WD supplemented with 10% fructooligosaccharides for 24 weeks. (b, c) Body weight curves for male mice fed WD + FOS show a pronounced reduction in weight gain in Adipoq‐F2‐KO mice compared to floxed controls from ~12 weeks onward, *n* = 6–7 per group. (d) Organ weights measured at study endpoint demonstrate significantly lighter livers and epididymal adipose depots in knockouts, *n* = 6–7 per group. (e, f) IPGTT after 8 weeks of WD + FOS feeding reveals no differences in glucose clearance, confirmed by area under the curve analysis. Data represent a pilot subset (*n* = 3), with full cohort data presented in (a–d) and (g–j). (g, h) IPITT indicates normal insulin sensitivity in both genotypes, confirmed by kITT calculations, *n* = 5 per group. (i, j) Plasma NEFAs following an 18 h fast and a subsequent glucose challenge show. no significant differences in lipolysis or insulin‐mediated suppression of lipolysis, *n* = 7 per group. Values are mean ± SEM; *p* < 0.05 considered significant.

To investigate the role of SCFAs acting via FFA2 to regulate metabolic outcomes (al Mahri et al., 2022; Kimura et al., 2020), we fed Adipoq‐F2‐KO and control mice WD supplemented with 10% FOS over 24 weeks. This diet is designed to increase plasma levels of SCFAs, particularly acetate, which binds with high affinity to FFA2. This experimental cohort aimed to assess the impact of FFA2 deletion in adipose tissue on metabolic responses in the presence of increased SCFA availability (Figure 3a).

Adipoq‐F2‐KO mice gained significantly less weight than their controls (Figure 3b,c) from week 12 through week 22 on the WD‐FOS diet (Table S1), with both genotypes plateauing after the 22nd week. In addition to reduced overall weight gain, Adipoq‐F2‐KO mice had significantly smaller livers and epididymal adipose depots than the controls at the end of the study (Figure 3d). These results suggest that adipose‐specific FFA2 KO may limit the ability to store fat under elevated SCFA levels.

Despite these differences in weight gain and fat storage, glucose homeostasis remained largely unaffected by the deletion of FFA2 in adipose tissue. IPGTT and IPITT were conducted after 8 weeks of diet exposure and showed no significant differences between the genotypes in glucose clearance (Figure 3e,f) and insulin sensitivity (Figure 3g,h). For reasons noted before, we next explored the effect of FFA2 on fasting‐induced lipolysis and observed no difference in plasma NEFAs or insulin secreted in response to the glucose challenge (Figure 3i,j). These data reveal that adipose‐specific FFA2 plays a role in modulating weight gain and fat storage in the presence of dietary fiber and SCFAs.

### 3.4 Adipoq‐F2‐KO mice exhibit No differences in energy expenditure or food intake on fiber supplemented Western diet

**FIGURE 4:**
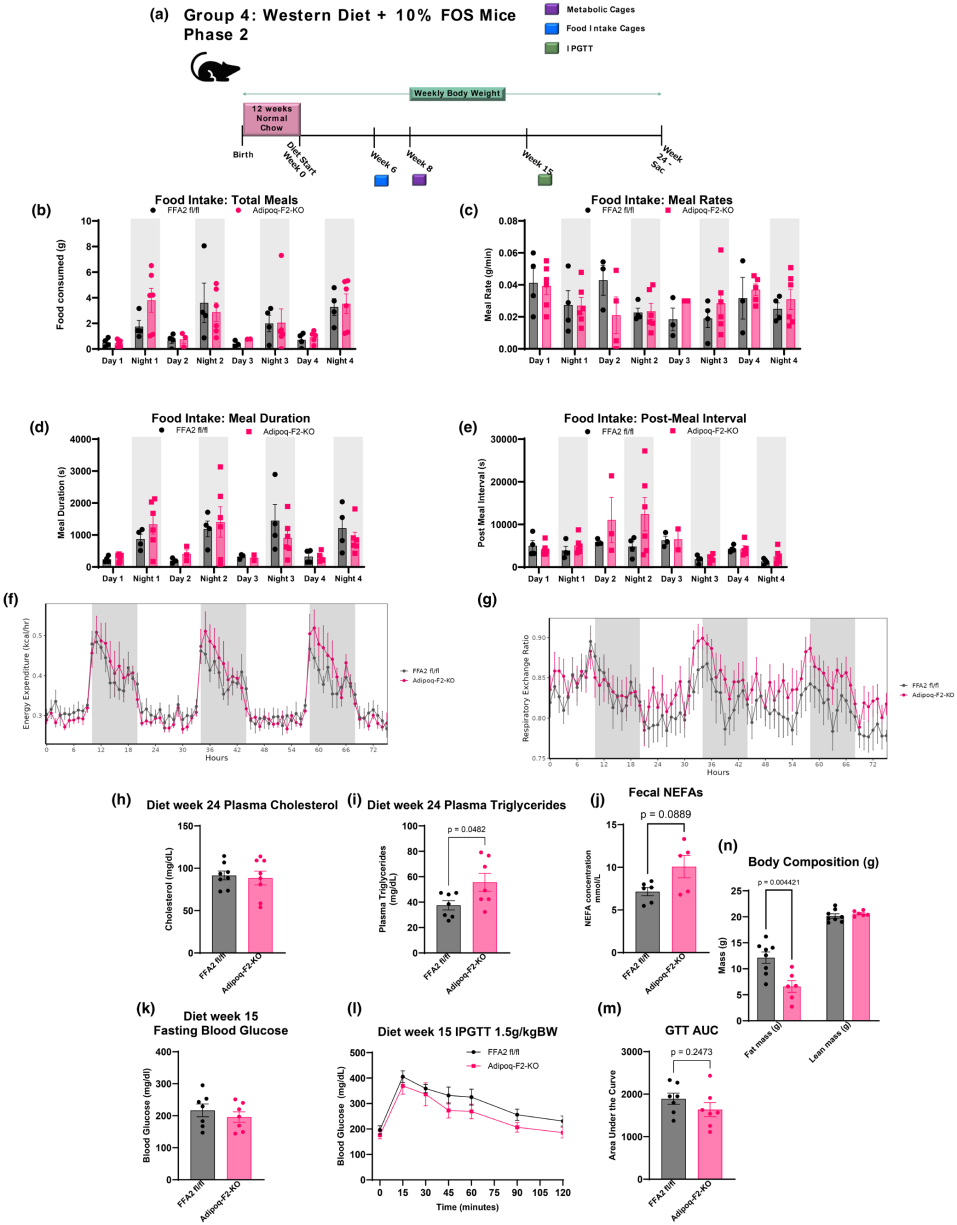
Reduced weight gain in Adipoq‐F2‐KO mice occurs despite comparable food intake and energy expenditure, pointing to impaired nutrient absorption. (a) Study timeline for indirect calorimetry, food‐intake measurements, and IPGTT in a separate WD + FOS cohort. (b–e) Detailed food‐intake metrics (total intake, meal size, meal duration, and time between meals) reveal no differences between genotypes, *n* = 4–6 per group. (f) Energy expenditure measured by indirect calorimetry (VCO_2_‐based + VO_2_‐based) shows no significant genotype effect based on area under the curve and ANCOVA analyses run separately for dark, light, and whole day period (statistical tables shown in supplementary data), *n* = 5–6 per group. (g) Respiratory exchange ratio (RER) trends higher (approaching 1.0) in Adipoq‐F2‐KO mice, suggesting a mild shift toward carbohydrate utilization, *n* = 5 per group. (h, i) Plasma cholesterol is unchanged, but plasma triglycerides are significantly higher in Adipoq‐F2‐KO, *n* = 6–7 per group. (j) Fecal NEFA content is elevated in knockout mice, hinting at impaired intestinal fat absorption, *n* = 5–6 per group. (k–m) IPGTT repeated later in the study confirms no difference in glucose clearance despite the emerging weight phenotype, *n* = 6–7 per group. (n) Body Composition measurements at 18 weeks of age, *n* = 8 per group. Data are mean ± SEM, *n* ≥ 6/group; statistical significance by Student's *t*‐tests or two‐way ANOVA as appropriate. Indirect calorimetry analyzed using CalR web application with embedded ANCOVA analysis. Area under the curve analyses and ANCOVA statistical tables in Figure S4 and Table S5, respectively.

We next sought to investigate whether differences in food intake, EE, or substrate utilization might explain the weight divergence between the Adipoq‐F2‐KO mice and their controls, which had previously become apparent after 12 weeks on the diet. Thus, a separate cohort of mice was again challenged with the WD‐FOS diet for 24 weeks. We, therefore, assessed the mice before the emergence of the weight differences to fully understand how adipose‐specific FFA2 deletion influenced metabolic parameters (Figure 4a). Food intake and energy expenditure were measured at weeks 6 and 8, respectively. These timepoints were selected to capture metabolic changes during the early phase of weight divergence rather than after the phenotype was fully established. We observed no food intake differences after 6 weeks of diet treatment between Adipoq‐F2‐KO and control mice. Also, the total number of meals consumed, the duration of each meal, post‐meal intervals, and meal speed were all comparable between the genotypes (Figure 4b–e). These data indicate that the reduced weight gain observed in the Adipoq‐F2‐KO mice cannot be attributed to differences in food consumption, suggesting that factors beyond overall caloric intake may be driving the weight divergence.

We next characterized both genotypes by indirect calorimetry, assessing oxygen consumption (VO2), carbon dioxide production (VCO2), respiratory exchange ratio (RER), and EE. Our data revealed no significant differences in VO2, VCO2,(Figure S3), or EE (Figure 4f) between Adipoq‐F2‐KO mice and controls, as determined by area under the curve (AUC) analysis and analysis of covariance (ANCOVA) across full day, dark, and light periods (Figure S3 and Table S2). While Adipoq‐F2‐KO mice displayed a modest increase in cumulative distance traveled within the cages (Figure S3j), this was not reflected in cumulative EE (Figure S3i), nor in the statistical analysis of locomotor parameters, which also remained non‐significant across full‐day and light/dark intervals (Table S2d; all *p* > 0.28). These findings indicate that locomotor activity does not contribute to the reduced adiposity at 23°C.

Additionally, there was a slight trend toward an increased RER in the Adipoq‐F2‐KO mice, particularly during the light/resting period, where the RER approached 1.0, suggesting a preference for carbohydrate metabolism over fat (Figure 4g). Although this trend did not reach statistical significance (*p* = 0.09 for the full day, *p* = 0.07 for the light period), it suggests a subtle shift in substrate utilization in Adipoq‐F2‐KO mice, with a greater reliance on glucose over fat as an energy source during rest periods. Given that food intake levels were similar between genotypes, but RER recordings indicated a trend toward decreased fat utilization in Adipoq‐F2‐KO mice, we next assessed circulating lipid levels to determine if there were differences in fat presence in the plasma, which could represent differences in fat clearance/storage in the adipocytes. While circulating cholesterol levels remained unchanged, plasma triglycerides were surprisingly significantly higher in Adipoq‐F2‐KO mice (Figure 4h,i), suggesting potential impairments in lipid metabolism or clearance.

We also measured fecal NEFA content from samples collected during the indirect calorimetry period to assess intestinal fat absorption. Adipoq‐F2‐KO mice tended to have higher fecal NEFA content than controls (Figure 4j), suggesting mildly impaired intestinal fat absorption that may contribute to the observed weight differences. The increase in plasma triglycerides in the knockout mice suggests a possible disruption in fat utilization or storage, potentially indicating a metabolic inefficiency in processing or storing fat as an energy substrate in the absence of FFA2.

In addition, glucose tolerance was consistently similar between Adipoq‐F2‐KO and control mice, even after divergence in weight gain (Figure 4k–m). Furthermore, fasting glucose levels were also comparable between the two groups (Figure 4k), suggesting that adipose‐specific FFA2 deletion does not significantly affect glucose handling, even after the mice developed distinct weight phenotypes.

In conclusion, despite the observed differences in weight gain between Adipoq‐F2‐KO and control mice on WD‐FOS diet, recapitulated in body composition measurements in this metabolic phenotyping cohort (Figure 4n), there were no significant differences in food intake, EE, or glucose tolerance to account for this divergence. The slight trend toward a higher RER in the Adipoq‐F2‐KO mice suggests a possible preference for carbohydrate metabolism, and this is supported by a trend toward increased fecal fat, i.e., decreased intestinal fat absorption. Together, these findings suggest that the decreased weight gain in Adipoq‐F2‐KO mice may be driven by mechanisms beyond basic energy balance or glucose homeostasis, likely involving altered fat absorption and storage efficiency in the absence of FFA2 signaling in adipose tissue.

### 3.5 Thermoneutrality highlights reduced fat mass and early weight divergence in adipoq‐F2‐KO mice despite comparable food intake and energy expenditure

**FIGURE 5:**
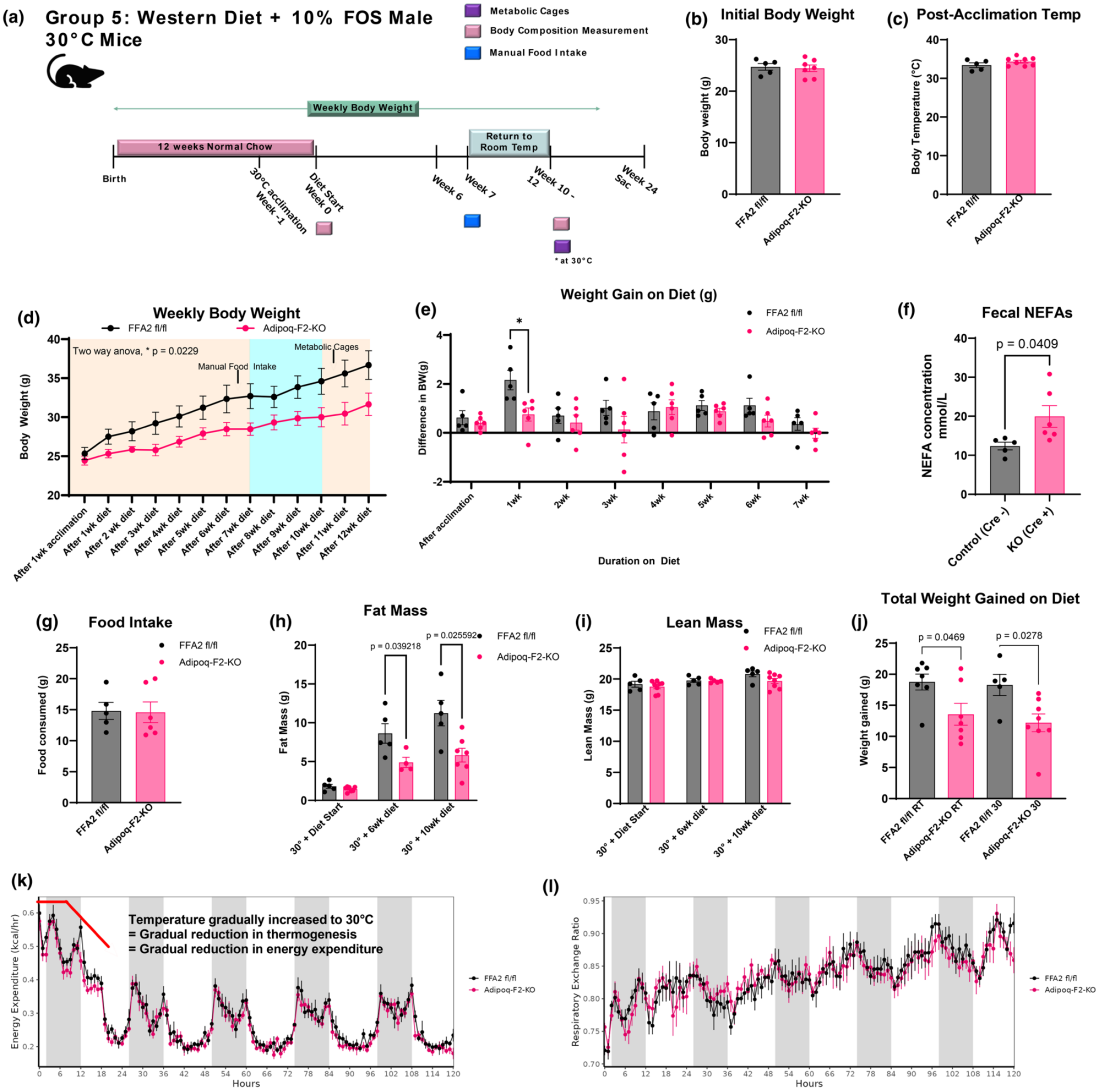
Thermoneutral conditions accentuate early weight divergence in Adipoq‐F2‐KO mice fed WD + FOS, yet food intake and energy expenditure remain comparable between genotypes. (a) Experimental design: At 11 weeks of age, mice receiving normal chow diet were transferred from room temperature housing to 30°C (thermoneutral) housing for a 1‐week acclimation period. Diet was then switched to WD + FOS for 24 weeks, with body composition measured at multiple time points. (b) Body weights were comparable between genotypes prior to initial acclimation at 30°C, *n* = 5–6 per group. (c) Post‐acclimation rectal temperatures were also comparable between genotypes, *n* = 5–6 per group. (d, e) Rapid and early weight divergence appears within the first week of WD + FOS in thermoneutral conditions and persists throughout the study, with Adipoq‐F2‐KO mice consistently lighter, *n* = 5–6 per group. (f) Fecal NEFAs are significantly elevated in Adipoq‐F2‐KO, consistent with impaired intestinal fat absorption, *n* = 5–6 per group. (g) Food intake measurements show no genotype difference, *n* = 5–6 per group. (h, i) Body composition indicates significantly lower fat mass in Adipoq‐F2‐KO by 6 weeks on WD + FOS at thermoneutrality, persisting into 10 weeks, confirming an inability to accumulate adipose tissue, *n* = 5–6 per group. (j) Total weight gained on diet comparing room temperature (RT, 22°C–23°C) and thermoneutral (30°C) housing conditions. Adipoq‐F2‐KO mice gained significantly less weight than controls at both temperatures, with comparable overall weight differences between genotypes despite earlier phenotype emergence at thermoneutrality, *n* = 5–7 per group. (k, l) Indirect calorimetry, upon shifting from room temperature up to 30°C, reveals no differences in energy expenditure or respiratory exchange ratio between genotypes (VCO_2_, VO_2_, locomotor activity shown in Figure S6), *n* = 5–6 per group. Values are mean ± SEM; *p* < 0.05 considered significant by two‐way ANOVA or *t*‐tests. Indirect calorimetry analyzed using CalR web application with embedded ANCOVA analysis. ANCOVA statistical tables in Table S7.

Noting that adipose‐specific metabolic responses can be obscured at room temperature due to the role of brown and beige adipose tissue in maintaining body temperature during mild cold stress (22°C) (McKie & Wright, 2021; Reitman, 2018), we next performed our studies at temperatures in the thermoneutral zone for mice (30°C). After a 1‐week acclimation period in 30°C housing, a separate cohort of mice was placed on a WD‐FOS diet, and metabolic parameters were measured over 24 weeks (Figure 5a). Before diet exposure, the transition to thermoneutral housing had no significant effect on weight gain in either genotype, as both groups gained weight at similar rates during the temperature acclimation period and maintained similar rectal temperatures (Figure 5b,c). However, after the WD‐FOS introduction, controls gained significantly more weight than the Adipoq‐F2‐KO mice, with the most pronounced weight gain occurring within the first week of diet exposure (Figure 5d,e). This early weight divergence was maintained throughout the study, with control mice consistently weighing more than the knockout group. Despite the substantial difference in weight gain, food intake did not differ between genotypes, indicating that the observed weight difference was not driven by initial caloric intake (Figure 5g). Further analysis of body composition revealed that the weight difference between the groups was primarily due to significantly reduced body fat in the Adipoq‐F2‐KO mice compared to controls (Figure 5h,i). Interestingly, although most of the weight difference manifested early, the overall weight differences after 24 weeks on the diet were similar to those observed in previous cohorts housed at room temperature (Figure 5j).

The earlier presentation of the weight difference for this cohort directly resulted from the combination of WD + FOS access and thermoneutral conditions. To further characterize this, we cycled both Adipoq‐F2‐KO mice and controls through a secondary temperature acclimation period where mice were temporarily returned to room temperature and then placed in indirect calorimetry housing that was gradually ramped up to thermoneutral temperatures and given access to the WD + FOS diet to allow us to capture the exact energetic changes that lead to the initial rapid weight gain and early manifestation of the weight difference. Metabolic measurements, including VO2, VCO2, and RER, were collected during this transition (Figure 5k,l and Figure S4). Our data, surprisingly, showed no significant differences in EE, VO2, VCO2, RER, or locomotor activity between the Adipoq‐F2‐KO and control mice (Table S3). These findings suggest that both genotypes engage in temperature regulation at similar rates. Additionally, these findings indicate that the reduced fat accumulation observed in the Adipoq‐F2‐KO mice results from other intrinsic metabolic differences.

Fecal samples collected during these metabolic measurements showed that Adipoq‐F2‐KO mice had significantly higher levels of fecal NEFAs (Figure 5f). These findings suggest that previously observed impaired intestinal fat absorption in Adipoq‐F2‐KO mice persists under thermoneutral conditions. Notably, the impairment becomes more readily apparent at thermoneutrality, suggesting that the mild cold stress experienced at room temperature may temporarily mask absorption differences.

This conclusion is further supported by a separate cold stress study (Figure S4), where exposure to lower temperatures failed to trigger the same weight divergence seen at room temperature or thermoneutrality. This outcome confirms that the leanness observed in Adipoq‐F2‐KO mice is not due to increased fat utilization for temperature regulation. Additionally, metabolic parameters remained consistent across multiple temperature phases, reinforcing the idea that the metabolic phenotype observed in Adipoq‐F2‐KO mice is due to factors beyond temperature‐related EE.

In summary, our experimental plan ensured that metabolic stressors did not obscure the observed phenotypes, indicating that the reduced fat accumulation in Adipoq‐F2‐KO mice appears to be driven by an intrinsic inability to absorb fat from ingested food and store absorbed fat rather than differences in food intake or EE.

### 3.6 Loss of adipose‐specific FFA2 drives impaired intestinal fat uptake via adipose‐intestinal crosstalk

**FIGURE 6:**
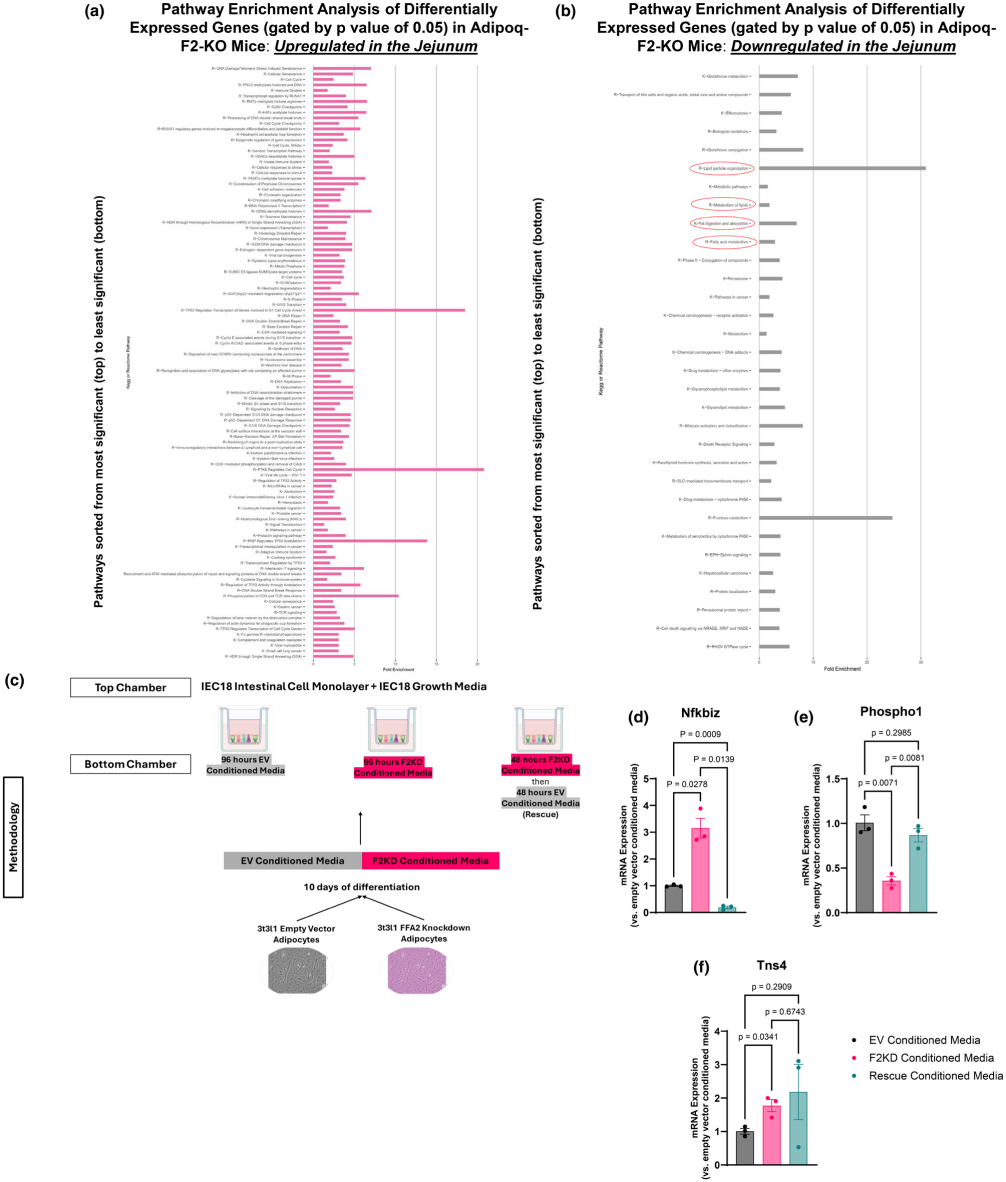
Loss of adipose FFA2 alters adipose‐intestinal crosstalk, leading to downregulated intestinal fat absorption pathways. (a) Pathway enrichment analysis based on differentially expressed genes from RNA‐Seq of jejunal mucosa collected after 2 weeks of WD + FOS diet treatment at thermoneutrality, *n* = 4 per group, identifies mild upregulation of immune pathways and cell stress. (b) Pathways involved in fat digestion, absorption, and metabolism are downregulated in Adipoq‐F2‐KO jejunum compared to floxed controls, *n* = 4 per group. (c) Transwell co‐culture setup: IEC18 intestinal epithelial cells in the top chamber were exposed to conditioned media from differentiated 3 T3‐L1 adipocytes (either empty vector [EV] or FFA2 knockdown [F2KD]). A “rescue” condition (F2KD‐conditioned medium replaced by EV medium halfway through the treatment period) was also tested. (d–f) qPCR of IEC18 cells after conditioned media treatment revealed altered expression of Nfkbiz, Phospho1, and Tns4 in cells treated with F2KD‐conditioned media compared to EV, partly rescued by switching to EV media. Data are mean ± SEM.

Increased fecal NEFA content in Figure 5 suggests that the absence of FFA2 in the adipose tissue may lead to altered adipokines or other potential mechanisms that impact intestinal fat uptake. To investigate this further, we performed RNA‐Seq on intestinal mucosa from the jejunum of both FFA2‐fl/fl and Adipoq‐F2‐KO mice after 2 weeks of WD + FOS diet treatment under thermoneutral conditions once the weight phenotype had emerged. We selected the jejunum for this analysis as it is the primary intestinal region responsible for dietary fat absorption. As expected, downregulated pathways in the Adipoq‐F2‐KO mice included fat digestion, absorption, and metabolism (Figure 6a,b), aligning with our previous observation that Adipoq‐F2‐KO mice had increased fecal NEFA content and agreeing with our hypothesis that the difference in weight between the two genotypes is likely due to decreased intestinal fat absorption when FFA2 is absent in the adipose tissue. Importantly, FFA2 expression in the jejunum was unchanged between genotypes (RNA‐seq data), confirming that the observed intestinal phenotype results from altered adipose‐derived signaling rather than direct effects on intestinal FFA2.

To determine whether this phenotype in Adipoq‐F2‐KO mice occurs due to crosstalk with adipose tissue, we utilized a transwell co‐culture system. CM collected from EV (3 T3‐L1 adipocytes transfected with empty vector) or F2KD cells after differentiation was used to treat IEC18 cells (a cell line generated from rat ileal cells) cultured in the upper chamber of a transwell system, allowing for the diffusion of adipocyte‐derived secreted factors. IEC18 cells were exposed to one of the following three conditions: (1) EV‐CM for 96 h, (2) F2KD‐CM for 96 h, or (3) F2KD‐CM for 48 h, followed by EV‐CM for the remaining 48 h (“rescue” condition) (Figure 6c). Following treatment, gene expression was examined in IEC18 cells by qPCR to compare with data obtained in the jejunal RNASeq. Interestingly, Nfkbiz, a regulator of inflammatory responses, was significantly upregulated in cells exposed to F2KD‐CM compared to EV‐CM (Figure 6d). This increase was reversed in the rescue condition, mirroring the RNA‐Seq data, where Nfkbiz was significantly increased in the jejunum of Adipoq‐F2‐KO mice. Additionally, Phospho1, a lipid metabolism regulator, was downregulated in F2KD‐CM treated cells compared to EV‐treated cells, but partially restored in the rescue condition, in line with RNA‐Seq data showing higher Phospho1 expression in the jejunum of control mice (Figure 6e). Finally, Tn3 was significantly upregulated in F2KD‐CM treated cells, but unlike the other genes, this increase was not reversed in the rescue condition (Figure 6f), suggesting a more persistent response to FFA2 loss. These findings suggest that adipose‐derived factors from FFA2‐deficient adipocytes alter intestinal gene expression, providing further evidence that adipose‐intestinal crosstalk contributes to the observed intestinal phenotype in Adipoq‐F2‐KO mice.

### 3.7 Adipoq‐F2‐KO mice exhibit increased inflammation and macrophage infiltration in epididymal fat, even under non‐inflammatory diet conditions

**FIGURE 7:**
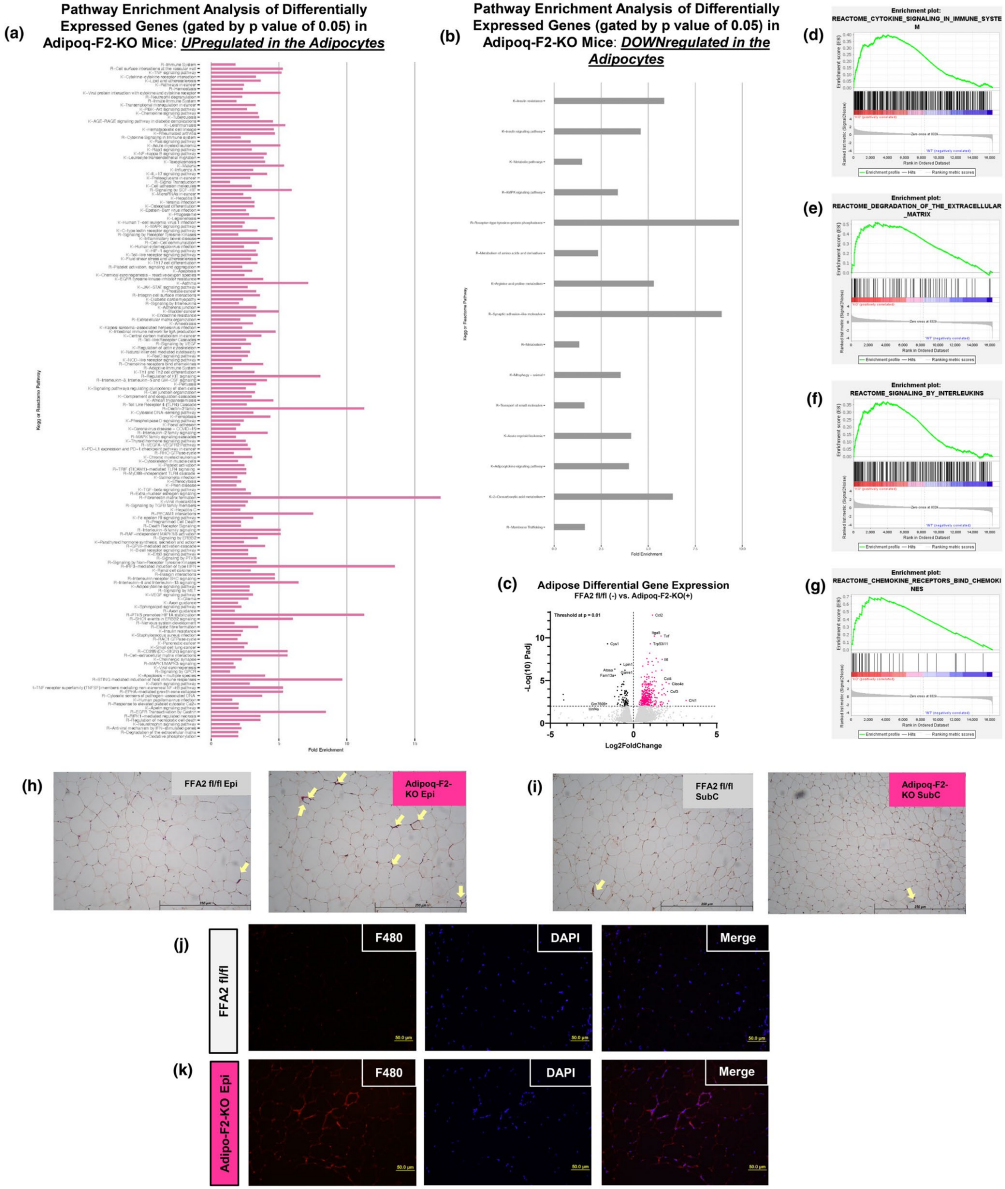
Adipose‐specific FFA2 deletion predisposes epididymal fat to inflammation and macrophage infiltration, even on a normal chow diet. (a) Pathway enrichment analysis based on differentially expressed genes from RNA‐Seq of mature epididymal adipocytes (adipocytes only) from Adipoq‐F2‐KO vs. floxed mice with normal chow/baseline diet identifies significant upregulation of immune and inflammatory pathways (cytokine–cytokine receptor interactions, interleukin signaling, ECM remodeling), *n* = 3–4 per group. (b, c) Control (floxed) adipocytes show relatively higher expression of metabolic and adipokine signaling genes (e.g., *Lpin1*, *Fam13a*), whereas knockout adipocytes show elevated pro‐inflammatory mediators (*Ccl2, Il6, Csf3*). (d–g) GSEA enrichment plots further illustrate robust immune activation in Adipoq‐F2‐KO adipose tissue. (h, i) H&E‐stained adipose sections reveal increased crown‐like structures in epididymal fat of Adipoq‐F2‐KO mice, with minimal changes in subcutaneous depots. Representative images from *n* ≥ 3 mice per group (Images from other mice in Figure S8). (j, k) F4/80 immunofluorescence confirms increased macrophage infiltration in epididymal fat from WD + FOS‐fed knockouts under thermoneutrality, corroborating the pro‐inflammatory transcriptomic signature across diets. Scale bars are 100 μm. Representative images from *n* ≥ 3 mice per group (Images from other mice in Figure S8).

From the jejunal RNA‐seq data, the pathway analyses suggest a reduced capacity for dietary fat processing and absorption in Adipoq‐F2‐KO mice. To understand the adipocyte changes that may be driving these intestinal changes, we performed RNA‐Seq analysis on mature white adipocytes isolated from epididymal fat pads of mice fed an SC diet. This approach enabled us to capture gene expression changes and assess how FFA2 knockout in adipocytes impacts metabolic pathways under baseline conditions. Our goal was to identify alterations in adipose gene expression, and by extension, the adipose secretome, that may contribute to the observed intestinal phenotype through inter‐tissue communication. Our RNA‐Seq analysis revealed that even when fed a non‐inflammatory SC diet, Adipoq‐F2‐KO mice exhibited elevated transcription of genes positively correlating with immune system mobilization, cytokine‐cytokine receptor interactions and signaling in the immune system, extracellular matrix degradation, signaling by interleukins, and chemokine signaling (Figure 7a,d–g). Enrichment plots (Figure 7d–g) generated through gene set enrichment analysis highlight these trends by ranking genes based on both the direction and magnitude of expression changes, where the enrichment score reflects the cumulative enrichment of genes in a given pathway across the ranked gene list, with positive scores indicating upregulation in Adipoq‐F2‐KO mice. These analyses demonstrate that genes associated with immune activation, cytokine signaling, and extracellular matrix remodeling are significantly enriched in the Adipoq‐F2‐KO group, underscoring that the absence of adipose‐specific FFA2 predisposes these mice to heightened intra‐depot inflammation despite the lack of WD to induce such stress responses. Notably, the control mice showed upregulation of genes involved in metabolic processes, including those associated with general metabolic pathways, insulin signaling, and adipocytokine (adipokine) signaling (Figure 7b), with key upregulated genes (higher fold change) in the control group including *LPIN1* (Lipin 1) and *FAM13A* (Family With Sequence Similarity 13 Member A), which encode proteins that regulate fat‐uptake, storage, and utilization, indicating that FFA2 may contribute to metabolic efficiency in adipocytes (Figure 7b), which is lost in Adipoq‐F2‐KO mice.

In contrast, the adipocytes from Adipoq‐F2‐KO mice showed enrichment of immune‐related pathways, with genes such as *CCL2* (C‐C Motif Chemokine Ligand 2), *CSF3* (Colony Stimulating Factor 3), *CCL4* (C‐C Motif Chemokine Ligand 4), *CHL1* (Close Homolog of L1), and *IL6* (interleukin 6) being among the most upregulated (Figure 7c), underscoring a shift toward inflammation and immune cell infiltration in adipose tissue. Differential regulation of selected genes from RNA‐seq was confirmed by qPCR (Figure S6). Further supporting these findings, H&E staining of adipose tissue sections from Adipoq‐F2‐KO mice exhibited a markedly higher prevalence of crown‐like structures (CLS), particularly in the epididymal fat depot, where inflammation tends to be more pronounced (Khanna et al., 2022) (Figure 7h). Interestingly, subcutaneous fat depots displayed minimal macrophage infiltration and a notable absence of CLS formation, indicating a depot‐specific inflammatory response in Adipoq‐F2‐KO mice (Figure 7i). This was further confirmed by F4/80 macrophage staining, which revealed a marked increase in macrophage presence within the epididymal fat depot of Adipoq‐F2‐KO mice under WD + FOS conditions at thermoneutrality, compared to controls (Figure 7j,k and Figure S5). This finding indicates that the heightened inflammatory state observed in Adipoq‐F2‐KO mice under NC conditions persists in the presence of dietary fiber supplementation and under thermoneutral conditions.

In summary, these data indicate that the loss of FFA2 in adipocytes drives a pro‐inflammatory phenotype in epididymal fat, characterized by increased macrophage infiltration and the formation of CLS, with gene expression levels changing even when mice are fed an NC diet. These effects persist under thermoneutral housing and WD‐FOS conditions, highlighting the role of FFA2 in maintaining adipose tissue homeostasis. Overall differential gene expression suggests that FFA2 in adipose tissue is critical for preventing inflammation and promoting metabolic efficiency in adipocytes.

### 3.8 FFA2 knockdown in 3 T3‐L1 cells increases ERK phosphorylation, reduces lipid storage, and sustains elevated CCL2 expression

**FIGURE 8:**
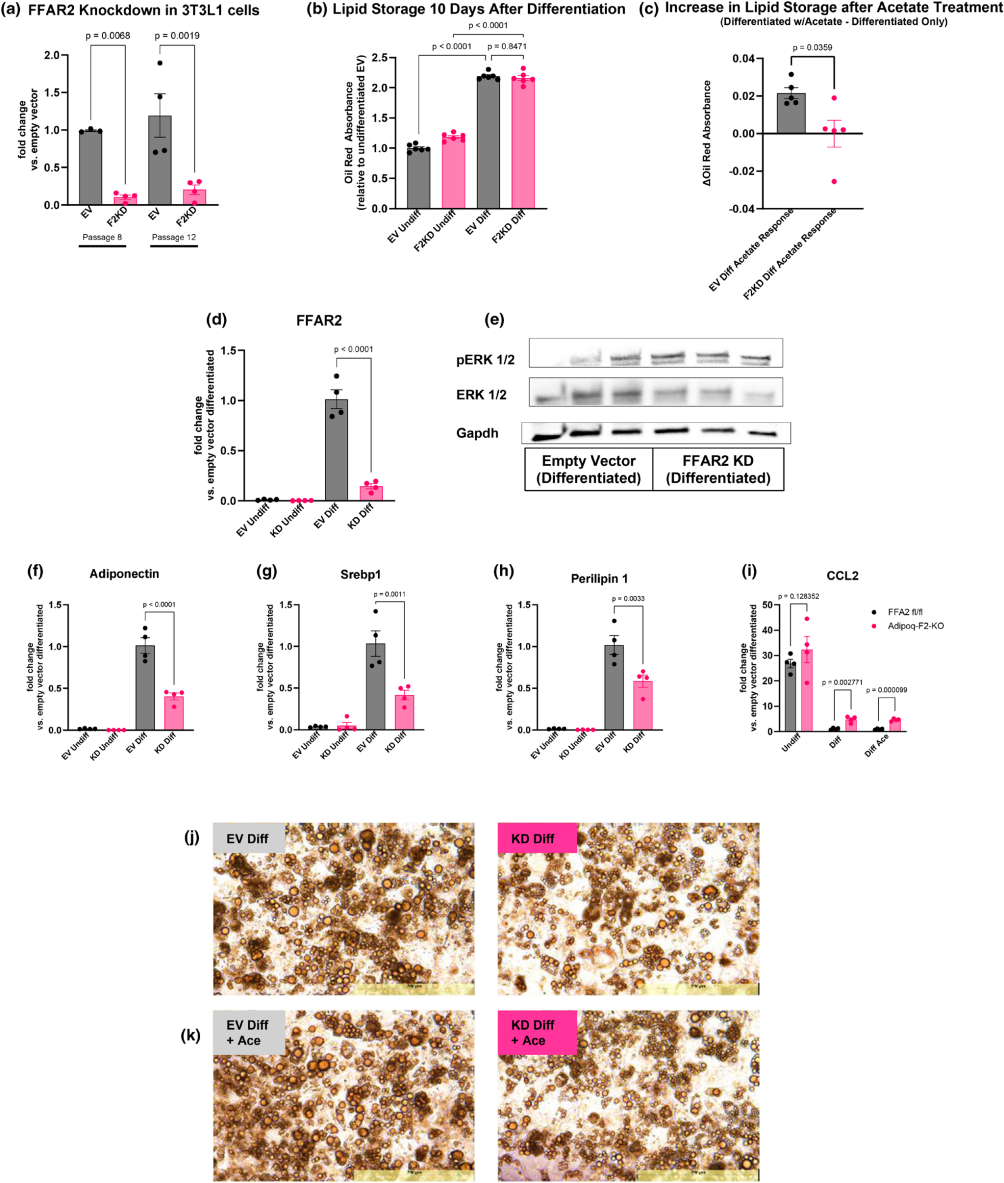
FFA2 knockdown in 3 T3‐L1 cells impairs long‐term lipid storage and maintenance of adipocyte maturity, increases ERK signaling, and sustains pro‐inflammatory gene expression. (a) Stable knockdown validation showing reduced FFA2 mRNA in F2KD vs. empty vector (EV) 3 T3‐L1 cells across multiple passages. (b) Oil Red O staining quantification after standard 10‐day differentiation showing that EV and F2KD cells initially differentiate to store the same amount of lipid. (c) Quantification of Oil Red O staining showing that F2KD cells fail to increase lipid storage in response to acetate treatment. (d) qPCR data showing that FFA2 mRNA expression increases drastically with adipocyte differentiation, likely indicating that this gene is necessary for maintaining adipocyte maturity. (e) Western blot in differentiated adipocytes reveals higher pERK1/2 levels in F2KD cells, indicating elevated MAPK signaling in the absence of FFA2. GAPDH serves as loading control. Complete Western blot with molecular weight markers is shown in Figure S9. The membrane was sequentially probed with anti‐pERK1/2, stripped and re‐probed with anti‐total ERK1/2, then stripped and re‐probed with anti‐GAPDH. (f–h) qPCR data showing reduced levels of *Adipoq*, *Srebp1*, *Plin1*, genes necessary for continued adipocyte maturity, in F2KD cells. (i) qPCR data showing persistently higher CCL2 expression in F2KD adipocytes. (j, k) Oil Red O staining of long‐term differentiation of EV and F2KD cells where EV cells store more lipid over a 30‐day differentiation period and this storage is increased in the presence of acetate. *N* = 3–4 wells per group for all experiments reported here. Data shown as mean ± SEM; *p* < 0.05 by *t*‐tests or one‐way ANOVA.

To assess the role of FFA2 in adipocyte differentiation and function, we used a stable 3 T3‐L1 F2KD cell line and EV controls. We confirmed the knockdown to be stable across multiple passages (Figure 8a). Both EV and F2KD cells were then differentiated into mature adipocytes using a cocktail containing insulin, dexamethasone, and IBMX. Successful differentiation was confirmed by lipid accumulation, visualized via Oil Red O staining. Lipid storage was comparable between EV and F2KD cells after 10 days under standard conditions during this initial differentiation phase (Figure 8b). However, when differentiation was repeated with the addition of 1 mM acetate, lipid storage significantly increased in EV cells but remained unchanged in F2KD cells, suggesting that FFA2 is necessary for acetate‐induced increase in adipocyte differentiation (Figure 8c).

While differentiation into mature adipocytes led to an overall rise in FFA2 expression for both genotypes, F2KD cells still exhibited significantly lower FFA2 levels compared to the EV cells, confirming the stability of the knockdown across developmental stages (Figure 8d). FFA2 knockdown also affected inflammatory signaling. During the differentiation process, EV cells showed a reduction in mRNA levels of inflammatory marker CCL2 (MCP1) (Ignacio et al., 2016) as they matured from preadipocytes to adipocytes (Figure 8i). In contrast, F2KD cells exhibited an incomplete reduction in CCL2 expression, both with and without acetate supplementation (Figure 8i). Furthermore, the rapid upregulation of FFA2 expression during differentiation (seen in control cells, Figure 8d) suggests that FFA2 contributes to adipocyte maturation and function. To assess long‐term effects of FFA2 knockdown beyond initial differentiation (during which both genotypes showed comparable lipid accumulation at day 10), EV and F2KD cells were maintained for 30 days post‐differentiation and then analyzed via oil red staining to evaluate long‐term lipid retention and adipocyte maintenance capacity. EV cells developed into larger, lipid‐filled adipocytes, while F2KD cells remained smaller and had less stored lipid content, indicating impaired adipocyte maintenance in the absence of FFA2 (Figure 8j and Figure S6). In addition, EV cells showed a marked increase in lipid storage after treatment with 1 mM acetate that was not observed in F2KD cells (Figure 8k).

Molecular analysis of differentiation confirmed that the knockdown of FFA2 reduced the expression of important adipocyte maturation genes, such as Adiponectin, SREBP1 (Sterol Regulatory Element‐Binding Protein 1), and PLIN1 (Perilipin 1) after 10 days of differentiation (Figure 8f–h). These reductions indicate that FFA2 plays a critical role in maintaining the mature adipocyte phenotype. The reduced fat storage in F2KD cells and the incomplete suppression of CCL2 highlight a dysfunctional adipocyte maturation process in the absence of FFA2, leading to impaired lipid handling and sustained inflammatory signaling. Additionally, western blot analysis of these differentiated cells revealed that ERK1/2 phosphorylation was significantly increased in F2KD cells compared to EV controls (Figure 8e). As ERK phosphorylation is a key regulatory mechanism during adipocyte maturation (American Diabetes Association, 2025; Donzelli et al., 2011; Gwon et al., 2013), the increased phosphorylation suggests that FFA2 may normally suppress ERK activity to facilitate proper adipocyte development. In addition to its role in maintaining adipocyte maturity, this finding suggests that FFA2 is required for the proper suppression of inflammatory signals during adipocyte maturation. The sustained elevation of CCL2 in F2KD cells may contribute to increased macrophage recruitment and inflammation in vivo, as elevated CCL2 levels are known to drive immune cell infiltration into adipose tissue (Lackey & Olefsky, 2016).

In summary, knockdown of FFA2 in 3 T3‐L1 cells disrupts adipocyte differentiation, impairs fat storage, and sustains elevated CCL2 expression, leading to heightened inflammatory responses. These data indicate that FFA2 is crucial for both the regulation of lipid metabolism and the suppression of inflammation during adipogenesis.

## 4 DISCUSSION

One suggested approach to managing metabolic health has focused on increasing dietary fiber intake due to its wide‐ranging benefits (Bulsiewicz, 2023), including reduced systemic inflammation, improved lipid metabolism, and a lower risk of metabolic diseases (Chen et al., 2020; Deehan et al., 2024; McRae, 2017). Historically, these benefits were attributed to fiber's physical properties, such as reduced caloric intake and increased intestinal transit time (Müller et al., 2018). However, recent studies have shown that many of these effects are mediated by GM, which ferments fiber into various byproducts, including SCFAs (Ang et al., 2018). These SCFAs exert myriad metabolic benefits (Mann et al., 2024) while also serving as ligands for receptors like FFA2, influencing immune responses and metabolic regulation. Given FFA2's role as an SCFA receptor, its activation in adipose tissue may be a key mechanism through which fiber exerts its metabolic benefits.

Some findings suggest that FFA2 promotes healthy adipose remodeling by enhancing adipogenesis and hyperplasia (Hong et al., 2005) while regulating controlled lipolysis (Ge et al., 2008). Some in vivo studies further support a protective metabolic role, as mice lacking FFA2 exhibited increased adiposity, while reintroducing FFA2 signaling led to weight reduction and improved insulin sensitivity (Kimura et al., 2013). Conversely, other studies suggest that FFA2 has no significant effect on glucose handling or adiposity (Zou et al., 2018). At the same time, some propose a detrimental role, showing that FFA2‐deficient mice were protected from weight gain, adiposity, and insulin resistance when fed a high‐fat diet (Bjursell et al., 2011; Brooks et al., 2017). Interestingly, most in vitro and ex vivo adipocyte‐focused studies indicate that FFA2 is essential for proper adipocyte function and generally exerts an anti‐inflammatory effect (Ge et al., 2008; Hong et al., 2005; Hu et al., 2016; Zaibi et al., 2010). These discrepancies suggest that the variability of in vivo results may be due to compensatory mechanisms or crosstalk from other organs, masking the direct effects of FFA2 on adipose tissue.

Our study directly addresses this challenge by utilizing an adipose‐specific KO mouse model (Adipoq‐F2‐KO), ensuring that any observed effects are specifically due to the absence of FFA2 in adipose tissue while eliminating confounding factors as in systemic KO models. This further allowed us to assess FFA2's local role and impact on adipose‐derived signaling to other tissues. Furthermore, our in vivo experiments included a fiber‐supplemented diet, where we could assess the role of FFA2 in the GM‐SCFA‐FFA2‐adipose signaling axis. This is a critical distinction from previous studies, which primarily used high‐fat diets and relied on acetate injections/water supplementation and synthetic agonists to activate FFA2 (Bjursell et al., 2011; Park et al., 2022; Priyadarshini et al., 2015; Schlatterer et al., 2021; Wada et al., 2025). While these methods ensure receptor activation, they bypass natural GM interactions with dietary fiber, potentially masking any feedback effects of adipose‐specific FFA2 on GM function. By preserving the complete GM‐SCFA‐FFA2 signaling pathway while selectively removing FFA2 in adipose tissue, our approach provides a more physiologically relevant model to understand its role in metabolic regulation.

Though our metabolic characterization of Adipoq‐F2‐KO mice on both SC and WD diets revealed no significant physiological differences compared to their floxed counterparts, the transcriptomic analysis revealed that Adipoq‐F2‐KO mice exhibit a predisposition to adipose inflammation even under SC conditions, suggesting an underlying inflammatory state despite the absence of overt metabolic dysfunction. We suspect that the apparent disconnect between transcriptional changes and physiological outcomes is likely due to the low metabolic stress associated with the SC diet. In addition, this diet treatment contains trace levels of fermentable fiber, resulting in low levels of SCFA production (and thus, FFA2 activation), leading to only mild receptor signaling in controls. In contrast, WD‐fed mice experience greater metabolic stress but lack any fiber‐derived SCFA signal, meaning FFA2 signaling does not impact the adipose tissues of both genotypes on this diet. These findings suggest that FFA2's role in adipose tissue becomes more pronounced in the presence of fiber‐derived SCFAs, with gene expression differences emerging under SC conditions but not translating into significant physiological changes until both WD‐induced metabolic stress and fiber exposure are introduced simultaneously.

Given the established roles of fiber in weight loss and metabolic syndrome alleviation, we initially hypothesized that the combination of metabolic stress from WD and active FFA2 signaling via WD‐FOS would lead to reduced weight and improved glycemic control in control mice compared to Adipoq‐F2‐KO mice, which we expected to be heavier and display signs of metabolic syndrome. Instead, we observed that Adipoq‐F2‐KO mice consistently weighed less and had lower fat mass than controls when fed a fiber‐supplemented WD. This suggests that FFA2 promotes overall adiposity without immediately observable effects on glycemic control. This unexpected finding led us to investigate food intake and EE, reasoning that FFA2 might influence adipokine signaling, which is known to alter hunger and satiety (Blüher, 2014; Clemente‐Suárez et al., 2023), where no differences were observed in these parameters. We measured food intake and EE during the early phase of weight divergence (weeks 6–8) to identify primary metabolic mechanisms, while later timepoint measurements could provide additional information about metabolic adaptations after substantial weight differences develop. As the plasma triglycerides in Adipoq‐F2‐KO mice were elevated, impaired lipid handling and adipose dysfunction in these mice may explain the significantly lighter weight than controls. This finding suggests that FFA2 may have a previously unrecognized role in systemic lipid metabolism, independent of its direct influence on weight or glycemic control. Future studies measuring LPL activity in adipose depots, hepatic VLDL secretion, and liver lipid content will help further elucidate the mechanisms underlying elevated plasma triglycerides and reduce liver mass in Adipoq‐F2‐KO mice. Additionally, further characterization of plasma glycerol levels across time will provide additional mechanistic insight into lipolytic regulation in Adipoq‐F2‐KO mice.

Given the established role of adipose tissue in temperature regulation in mice (Bastías‐Pérez et al., 2020; Ye et al., 2013) and prior studies showing that FFA2‐deficient mice exhibit higher core body temperatures (Bjursell et al., 2011), we conducted the remainder of our in vivo studies under thermoneutral housing conditions, reasoning that FFA2 might also influence temperature regulation, thereby increasing adipose tissue metabolic activity. Our rationale was that the observed weight differences could simply result from Adipoq‐F2‐KO mice diverting more ingested nutrients toward thermogenesis at room temperature, representing mild cold stress for mice. Notably, the weight divergence between genotypes only became significant around week 12 when mice were housed at room temperature and fed WD + FOS, suggesting that differences in EE may have been too subtle to detect during the 72‐h indirect calorimetry recording. However, the weight difference emerged immediately under thermoneutral conditions despite food intake and EE remaining unchanged. This led us to explore energy excretion as a potential contributor to the differences in fat mass. Indeed, fecal NEFA measurements revealed that Adipoq‐F2‐KO mice excreted 50% more NEFAs (a key energetic substrate derived from dietary fat) than controls, suggesting impaired intestinal lipid absorption as a driving factor in their reduced fat mass.

One possible explanation is that differences in intestinal lipid absorption are masked at room temperature due to the continuous low‐grade stress response that mice experience under these conditions. It is well established that housing mice at sub‐thermoneutral temperatures triggers chronic catecholamine secretion (Hylander et al., 2017; Hylander et al., 2019). In addition to their thermogenic effects via β‐adrenergic receptor activation, catecholamines reduce gut motility and prolong digestion by keeping the body in a chronic “fight‐or‐flight” state. Given this, a deficit in intestinal lipid absorption may take longer to detect under cold stress conditions, as digested materials remain in the intestinal lumen for an extended period, allowing more time for lipids to be absorbed into the circulation, processed, and stored. However, the chronic catecholamine response is alleviated at thermoneutrality, leading to increased gut motility. As a result, any deficiencies in lipid absorption would become immediately apparent, as the slowing effect is no longer present to retain fat in the intestinal lumen for prolonged absorption. Future studies will explore this hypothesis by analyzing the rate of phenotype emergence across a temperature gradient, measuring catecholamine levels at different temperatures, and assessing gut transit time to determine whether intestinal lipid absorption is differentially regulated under varying thermal conditions.

A jejunal RNA‐Seq analysis was conducted to investigate the possible mechanisms causing differential lipid absorption. Initial analysis based on individual gene expression differences, with FDR <0.05, showed no pathway enrichment, but as we were specifically testing for pathways in lipid metabolism to explain the 50% reduction in fatty acid absorption, we determined that it was appropriate to base pathway analysis on *p*‐value <0.05 alone. This reduced stringency revealed significant downregulation of pathways involved in fat digestion, absorption, and metabolism in Adipoq‐F2‐KO mice compared to controls when placed in thermoneutral housing conditions and at the point of weight divergence on a WD + FOS diet, supporting our hypothesis that loss of FFA2 in adipose tissue disrupts lipid processing in the intestines. Further, as the individual differential expression of genes in these pathways is less than a log‐2 fold difference, the phenotypic effect appears to be driven by a combination of more minor changes to overall intestinal function rather than the consequence of changes to only one or two of the major intestinal fat transporters. Finally, FFA2 and FFA3 gene expression in the jejunum remained unchanged between both genotypes, reinforcing the idea that the observed intestinal alterations were driven by disrupted crosstalk between adipose tissue (the site of the FFA2 knockout) and the intestine rather than a direct compensatory effect of FFA2 or FFA3 (the sister receptor) within the gut itself.

To establish a mechanism for adipose‐derived signaling driving intestinal function in this model, we then performed RNA‐Seq on selectively isolated mature white adipocytes to minimize gene expression signals from non‐adipocyte cell types such as adipose‐resident macrophages, which are not directly affected by the Adipoq‐F2‐KO model. This approach also allowed us to focus specifically on transcriptional changes occurring within the lipid‐storing and endocrine‐active compartments of adipose tissue, focusing on key pathways that may be responsible for the observed intestinal phenotype in Adipoq‐F2‐KO mice. Pathway analysis using the differentially expressed genes revealed that genes upregulated in Adipoq‐F2‐KO adipocytes were highly enriched for processes linked to immune system activation, TNF signaling, cytokine and chemokine signaling, and pro‐inflammatory pathways. These findings indicate that losing FFA2 in adipocytes promotes a low‐grade inflammatory state within the adipose tissue itself. Notably, TNF signaling was one of the most significantly upregulated pathways, aligning with prior studies demonstrating that inflammatory signaling from dysfunctional adipose tissue can contribute to metabolic dysregulation and systemic inflammation.

The increased expression of chemokines and cytokines suggests that adipose tissue in Adipoq‐F2‐KO mice may be actively recruiting immune cells, where our data show an apparent increase in macrophage presence in the epididymal depots of Adipoq‐F2‐KO mice in comparison to controls on the WD‐FOS diet. Overall, the pathway analysis suggests a chronic inflammatory state. Given that pro‐inflammatory cytokines such as TNF‐α, IL‐6, and CCL2 are known mediators of metabolic dysfunction and insulin resistance, their increased presence in FFA2‐deficient adipose tissue could be disrupting normal adipose‐intestinal communication, potentially explaining the heightened inflammatory and DNA damage‐related pathways observed in the jejunum of Adipoq‐F2‐KO mice.

Conversely, pathways downregulated in Adipoq‐F2‐KO adipocytes were strongly associated with metabolic regulation. Of particular interest was the downregulation of adipocytokine signaling pathways, which play a crucial role in maintaining whole‐body metabolic homeostasis and would be responsible for signaling to peripheral tissues such as the intestines. The reduced expression of genes involved in adipocyte function suggests that adipocytes in FFA2‐deficient mice exhibit signs of metabolic dysfunction, consistent with prior research highlighting the role of FFA2 in maintaining adipose tissue homeostasis.

These findings were further tested in vitro experiments where 3T3L1 adipocytes with FFA2‐KD showed a marked decrease in genes necessary for adipocyte maturity, normal metabolism, and general function, as well as reduced capacity for lipid storage after long‐term differentiation compared to control cells. Notably, these genes include well‐established PPARγ targets, suggesting that FFA2 deficiency may disrupt the PPARγ‐driven adipocyte maturation program; however, further experimentation is required to confirm this as a future direction for this study. Additionally, 3T3L1 adipocytes with intact FFA2 showed increased lipid storage in response to acetate, which was not observed in the cells with FFA2 deletion. Future characterization will involve direct triglyceride measurements in cell lysate and conditioned media to confirm the visual phenotype observed across these experimental replicates.

This apparent inability to maintain a mature adipocyte phenotype without FFA2 could explain disruptions in adipocytokine signaling. Combined with the increased expression of CCL2 in FFA2‐KD 3T3L1 adipocytes, these data suggest that FFA2 function may protect from inflammation and help maintain normal adipocyte metabolic function. Furthermore, the increased phosphorylation of ERK1/2 in the absence of FFA2 aligns with multiple previous reports where ERK1/2 activation has been shown to lead to broad metabolic dysfunction in adipocytes.

With resulting macrophage recruitment, the concurrent downregulation of adipocyte metabolic genes and upregulation of inflammatory pathways raises the possibility that adipocytes in Adipoq‐F2‐KO mice are transitioning into a dysfunctional state. This shift aligns with prior findings demonstrating that inflamed adipose tissue releases factors that negatively impact lipid handling in peripheral tissues, including the intestine. Given the observed intestinal lipid absorption deficits in Adipoq‐F2‐KO mice, it is plausible that the loss of FFA2 in adipose tissue disrupts adipocyte‐derived regulatory signals, leading to compromised lipid metabolism in the gut.

These findings provide compelling evidence that the inflammatory signature emerging in FFA2‐deficient adipocytes may be the key driver of the intestinal phenotype observed in Adipoq‐F2‐KO mice. The heightened immune activation and cytokine signaling in adipose tissue strongly parallel the inflammatory and DNA damage‐related pathways upregulated in the jejunum, suggesting that a pro‐inflammatory adipokine or signaling factor may be linking adipose dysfunction to intestinal pathology. Additionally, our in vitro experiments demonstrated that FFA2 knockdown in adipocytes led to impaired lipid storage and metabolic dysfunction, reinforcing the role of FFA2 in maintaining adipocyte health. These findings indicate that adipose‐FFA2 is a novel regulator of adipose‐gut communication with significant implications for metabolic disease. Multi‐omic profiling of CM from F2KD and control adipocytes and serum from Adipoq‐F2‐KO and control mice is underway to identify secreted factors contributing to this novel FFA2‐dependent adipose‐gut axis.

### 4.1 Limitations

Multiple limitations should be noted. (1) Our study tested the effects of fiber supplementation (10% FOS) specifically in the context of Western diet feeding, which represents the physiologically relevant scenario for therapeutic fiber interventions in obesity. However, we did not include a fiber‐supplemented standard chow group, which limits our ability to determine whether the observed phenotypes require the metabolic stress of high‐fat feeding or whether fiber supplementation alone would be sufficient to reveal FFA2‐dependent effects. Future studies examining fiber supplementation across expanded dietary backgrounds would help clarify this question. (2) While we examined lipolysis through measurement of serum and fecal NEFAs, we did not directly assess de novo lipogenesis in vivo. Given the reduced expression of lipogenic genes such as Srebp1 in FFA2‐deficient adipocytes (Figure 8g), impaired de novo lipogenesis may contribute to the reduced adipose mass and altered lipid metabolism observed in Adipoq‐F2‐KO mice. Future studies using isotopic tracers to measure lipogenic flux in vivo would help clarify the relative contributions of altered lipogenesis versus lipolysis to the metabolic phenotype. (3) And finally, our transwell co‐culture experiments used rat‐origin IEC18 intestinal epithelial cells with mouse‐origin 3 T3‐L1 adipocytes. While species‐matched cells or primary mouse intestinal organoids would be preferable, the conserved nature of the signaling pathways studied and the focus on secreted factors support the validity of this proof‐of‐concept approach. Future studies should validate these findings using mouse intestinal epithelial cells or ex vivo intestinal tissue.

### 4.2 Relevance to human health and therapeutic potential

These findings have important implications for human health, given the strong association between adipose dysfunction and metabolic diseases. Adipose inflammation has been linked to insulin resistance, dyslipidemia, and intestinal dysfunction, all of which are key contributors to obesity‐related metabolic disorders. Identifying adipose‐derived inflammatory signals that disrupt intestinal lipid metabolism suggests a potential therapeutic target for metabolic syndrome. Modulating adipose inflammation or restoring FFA2 function could help normalize gut‐adipose crosstalk, improving metabolic outcomes in individuals with obesity and metabolic dysfunction. Finally, these data raise the question of whether some of the beneficial effects of fiber are mediated at the adipose tissue level via FFA2.

## AUTHOR CONTRIBUTIONS

**Chioma Nnyamah:** Conceptualization; data curation; formal analysis; investigation; methodology; project administration; software; supervision; validation; visualization. **Brian T. Layden:** Conceptualization; funding acquisition; methodology; resources; supervision. **Medha Priyadarshini:** Formal analysis; methodology; validation; visualization. **Kai Xu:** Formal analysis; visualization. **Barton Wicksteed:** Conceptualization; data curation; formal analysis; investigation. **Nupur Pandya:** Methodology. **Laura J. Den Hartigh:** Methodology; validation; visualization. **James Boyett:** Conceptualization; formal analysis; investigation; methodology; visualization. **Irene Corona‐Avila:** Formal analysis; methodology. **Nadia Sweis:** Methodology; project administration. **Marissa St. George:** Methodology. **Abeer M. Mahmoud:** Methodology. **Jose Cordoba‐Chacon:** Conceptualization; data curation; methodology. **Yuwei Jiang:** Methodology.

## DATA VAILABILITY STATEMENT

The RNA sequencing datasets generated in this study have been deposited in the NCBI Gene Expression Omnibus (GEO) and are publicly accessible under accession numbers GSE302985 (adipocyte RNA‐seq) and GSE309021 (jejunum RNA‐seq). All other data supporting the findings of this study are available from the corresponding author upon reasonable request.

## ETHICS STATEMENT

This study was conducted in accordance with institutional, national, and ethical guidelines for the care and use of laboratory animals. All animal procedures were reviewed and approved by the Institutional Animal Care and Use Committee at the University of Illinois at Chicago. The study was designed to minimize animal distress, ensure appropriate housing and monitoring, and use scientifically justified sample sizes for metabolic, histological, transcriptomic, and cellular analyses. No human participants or human‐derived materials were used in this study.

## Supporting information


**Figure S1:** Adipoq‐F2‐KO Female Mice are metabolically comparable to floxed controls on a standard laboratory chow diet and SVF profiling across adipose depots indicates that FFA2 deletion occurs selectively in mature adipocytes. (a) Schematic of experimental timeline. (b) 10‐week body composition measurement showing no difference in either lean mass or fat mass between the two genotypes, *n* = 3–5 per group. (c, d) No observed differences in insulin sensitivity as measured by an ITT, *n* = 3–5 per group. (e–g) No observed differences in glucose tolerance as measured by a GTT or in fasting glucose levels. (h–l) FFA2 mRNA expression levels in the stromal vascular fractions across the 5 major adipose depot types confirm that the knockdown for this model primarily affects mature adipocytes. (m) qPCR measurement of FFA2 mRNA in whole tissue from perirenal fat, mesenteric fat, and interscapular brown adipose tissue (iBAT) showing reduced expression in Adipoq‐F2‐KO mice compared to highly variable levels in controls, *n* = 3–4 per group. Data are presented as mean ± SEM; statistical significance was assessed by two‐way ANOVA (for time courses) or Student's *t*‐tests (for single time points), with *p* < 0.05 considered significant.

**Figure S2:** Adipoq‐F2‐KO Female Mice are metabolically comparable to floxed controls on Western Diet. (a) Schematic of experimental timeline. (b) Weekly body weights measured over 12 weeks of diet treatment showing no difference in weight gain between the two genotypes, *n* = 5–6 per group. Adipoq‐F2‐KO mice had similar body composition to floxed controls before diet start (c) and they remained comparable after 12 weeks on WD (f), n = 5–6 per group. There were also no differences in insulin sensitivity measured by ITT (d, e), glucose tolerance measured by GTT (h, i), and fasting blood glucose (j), *n* = 5–6 per group. Adipoq‐F2‐KO mice gained significantly less weight than male counterparts after 12 weeks of diet exposure. Data are presented as mean ± SEM; statistical significance was assessed by two‐way ANOVA (for time courses) or Student's *t*‐tests (for single time points), with *p* < 0.05 considered significant.

**Figure S3:** Adipoq‐F2‐KO male mice on WD + FOS are comparable to floxed controls in terms of energy expenditure at room temperature. (a) Carbon dioxide produced (b) Oxygen consumed and (c) Locomotor Activity do not differ between Adipoq‐F2‐KO mice and floxed controls as measured by indirect calorimetry gas exchange systems. Apparent gaps between groups are not significant as measured by area under the curve analysis for (d–e) energy expenditure broken down by recording period (whole day, nightly) or (f–h) Respiratory Exchange Ratio broken down by recording period (whole day, daily, nightly). Cumulative Energy Expenditure (i) and Total Distance in cage (j) also show no difference. *n* = 5–6 per group. Data are presented as mean ± SEM; statistical significance was assessed by two‐way ANOVA (for time courses) or Student's *t*‐tests (for single time points), with *p* < 0.05 considered significant.

**Figure S4:** Adipoq‐F2‐KO male mice on WD + FOS are comparable to floxed controls in terms of energy expenditure at thermoneutrality and Adipoq‐F2‐KO mice remain comparable to floxed controls during cold exposure. (a) Carbon dioxide produced (b) Oxygen consumed and (c) Locomotor Activity do not differ between Adipoq‐F2‐KO mice and floxed controls as measured by indirect calorimetry gas exchange systems. (d) Experimental timeline illustrating dietary interventions (WD or WD + FOS) followed by acute cold exposure (4°C) initiated after 6 weeks of dietary challenge. (e) Adipoq‐F2‐KO mice exhibit comparable weight loss to floxed controls following 1 week of cold exposure. (f) Body composition analysis reveals no differences between Adipoq‐F2‐KO and floxed control mice prior to cold exposure. (g, h) Hourly measurements of body weight and core body temperature during cold acclimation. (i) Hourly weight loss during cold acclimation is comparable between groups. (j) Ad libitum blood glucose concentrations remain similar after cold exposure. (k) Body composition measurements after 1 week of cold exposure show no differences between Adipoq‐F2‐KO and floxed controls. Data presented as mean ± SEM; statistical significance assessed by two‐way ANOVA (for time courses) or Student's *t*‐test (for single time points), with *p* < 0.05 considered significant.

**Figure S5:** (a) Oil Red O staining of 3T3L1 EV and F2KD cells after 30 days of differentiation in the presence or absence of 1 mM acetate. (b) Complete Western blot image showing total ERK1/2 detection with molecular weight ladder (Bio‐Rad Precision Plus Protein Dual Color Standards, cat# 1610374). The membrane shown was first probed with anti‐pERK1/2, then stripped and re‐probed with anti‐total ERK1/2 (image shown), and subsequently stripped and re‐probed with anti‐GAPDH (not shown). ERK1/2 proteins migrate at their expected molecular weights of 42/44 kDa, appearing as a doublet between the 37 kDa and 50 kDa ladder markers. GAPDH (37 kDa) migrates at the corresponding ladder position. Molecular weight markers are labeled in kDa. This blot corresponds to Figure 8e in the main manuscript.

**Figure S6:** (a–e) qPCR validation of RNASeq gene expression. (f–i) H&E‐stained adipose tissue showing increased macrophage infiltration in Epididymal fat of Adipoq‐F2‐KO mice. (j–l) F480 macrophage marker immunofluorescence quantification (M‐N) F480 macrophage marker staining confirming increased macrophage presence. *N* = 3 per group. Data presented as mean ± SEM; statistical significance assessed by Student's *t*‐test with *p* < 0.05 considered significant.

**Table S1:** Statistical tables showing that Male Adipoq‐F2‐KO gain significantly less weight than floxed controls when given Western diet supplemented with 10% FOS at Room Temperature with the weight divergence being significant from week 12 through week 22 when mice are treated for 24 weeks.

**Table S2:** Adipoq‐F2‐KO Male Mice on WD + FOS are comparable to floxed controls in terms of energy expenditure at Room Temperature (continued). Tables present *p* values from ANCOVA using total mass (a), lean mass (b), and fat mass (c) as covariates, confirming no effect of mass on energy expenditure parameters as recorded. *N* = 5–6 per group with *p* < 0.05 considered significant. Tables present p values from ANOVA of mass‐independent variables (d) over recording timecourse.

**Table S3:** Adipoq‐F2‐KO Male Mice on WD + FOS are comparable to floxed controls in terms of energy expenditure at Thermoneutrality (continued). Tables present *p* values from ANCOVA using total mass (a), lean mass (b), and fat mass (c) as covariates, confirming no effect of mass on energy expenditure parameters as recorded. *N* = 5–6 per group with *p* < 0.05 considered significant. Tables present *p* values from ANOVA of mass‐independent variables (d) over recording timecourse.
