# Supplementary material for: Adipocyte‐specific FFA2 deletion leads to increased adipose inflammation and is associated with altered intestinal lipid handling in mice
Source: Physiol Rep. 2026 May 4;14(9):e70875. doi: 10.14814/phy2.70875 (PMC13139770; doi:10.14814/phy2.70875)
Supplement: Supplementary file 1 — Figure S1: Adipoq‐F2‐KO Female Mice are metabolically comparable to floxed controls on a standard laboratory chow diet and SVF profiling across adipose depots indicates that FFA2 deletion occurs selectively in mature adipocytes. (a) Schematic of experimental timeline. (b) 10‐week body composition measurement showing no difference in either lean mass or fat mass between the two genotypes, n = 3–5 per group. (c, d) No observed differences in insulin sensitivity as measured by an ITT, n = 3–5 per group. (e–g) No observed differences in glucose tolerance as measured by a GTT or in fasting glucose levels. (h–l) FFA2 mRNA expression levels in the stromal vascular fractions across the 5 major adipose depot types confirm that the knockdown for this model primarily affects mature adipocytes. (m) qPCR measurement of FFA2 mRNA in whole tissue from perirenal fat, mesenteric fat, and interscapular brown adipose tissue (iBAT) showing reduced expression in Adipoq‐F2‐KO mice compared to highly variable levels in controls, n = 3–4 per group. Data are presented as mean ± SEM; statistical significance was assessed by two‐way ANOVA (for time courses) or Student's t‐tests (for single time points), with p < 0.05 considered significant. [file PHY2-14-e70875-s002.pdf]

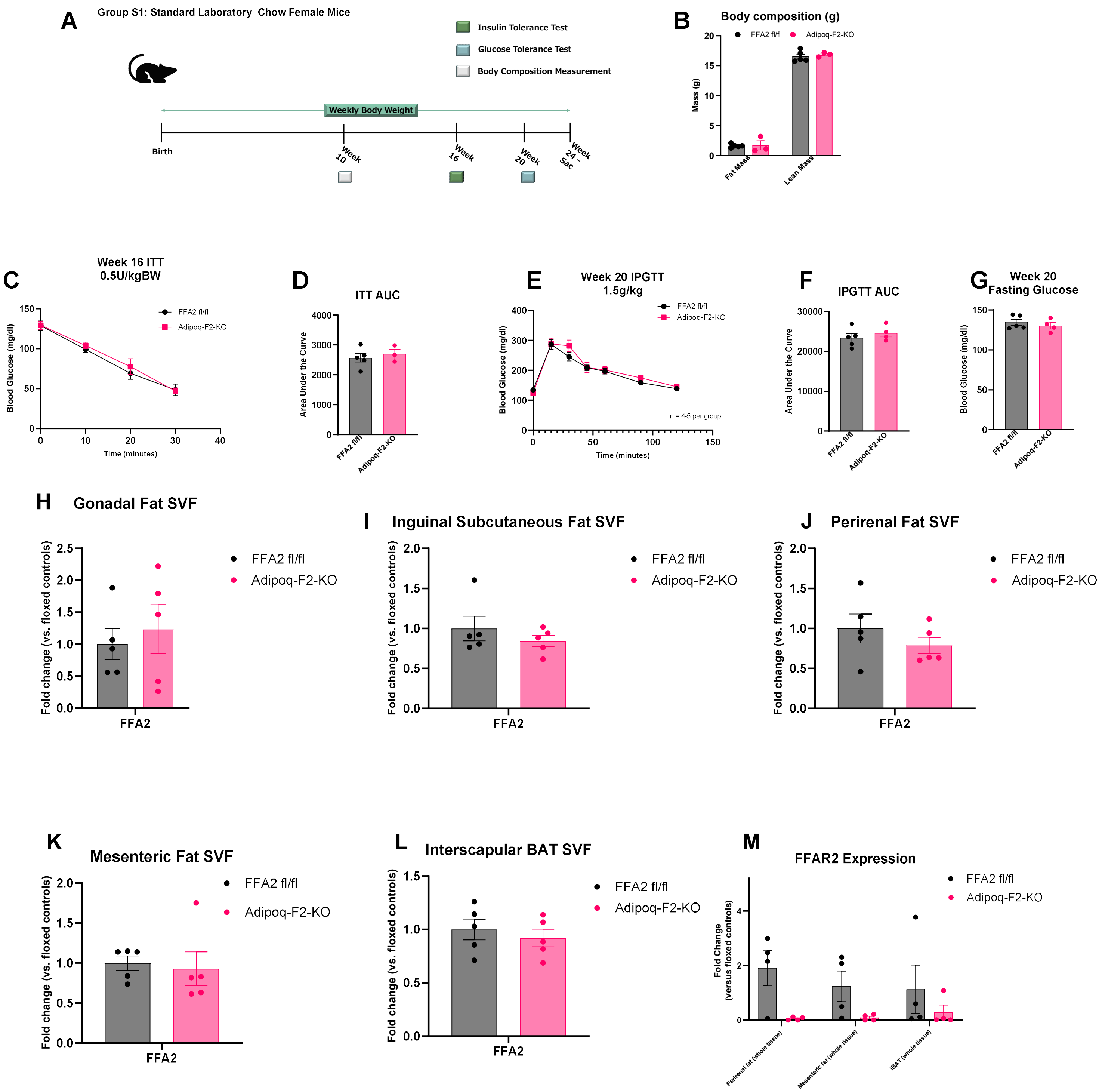

Supplementary Figure 1: Adipoq-F2-KO Female Mice are metabolically comparable to floxed controls on a Standard Laboratory Chow diet and SVF Profiling Across Adipose Depots Indicates That FFA2 Deletion Occurs Selectively in Mature Adipocytes.
(A) Schematic of experimental timeline. (B) 10-week body composition measurement showing no difference in either lean mass or fat mass between the two genotypes, n=3-5 per group. (C-D) No observed differences in insulin sensitivity as measured by an ITT, n=3-5 per group. (E-G) No observed differences in glucose tolerance as measured by a GTT or in fasting glucose levels. (H-L) FFA2 mRNA expression levels in the stromal vascular fractions across the 5 major adipose depot types confirm that the knockdown for this model primarily affects mature adipocytes. (M) qPCR measurement of FFA2 mRNA in whole tissue from perirenal fat, mesenteric fat, and interscapular brown adipose tissue (iBAT) showing reduced expression in Adipoq-F2-KO mice compared to highly variable levels in controls, n = 3-4 per group. Data are presented as mean ± SEM; statistical significance was assessed by two-way ANOVA (for time courses) or Student's t-tests (for single time points), with $p < 0.05$ considered significant.
