# Supplementary material for: Adipocyte‐specific FFA2 deletion leads to increased adipose inflammation and is associated with altered intestinal lipid handling in mice
Source: Physiol Rep. 2026 May 4;14(9):e70875. doi: 10.14814/phy2.70875 (PMC13139770; doi:10.14814/phy2.70875)
Supplement: Supplementary file 2 — Figure S2: Adipoq‐F2‐KO Female Mice are metabolically comparable to floxed controls on Western Diet. (a) Schematic of experimental timeline. (b) Weekly body weights measured over 12 weeks of diet treatment showing no difference in weight gain between the two genotypes, n = 5–6 per group. Adipoq‐F2‐KO mice had similar body composition to floxed controls before diet start (c) and they remained comparable after 12 weeks on WD (f), n = 5–6 per group. There were also no differences in insulin sensitivity measured by ITT (d, e), glucose tolerance measured by GTT (h, i), and fasting blood glucose (j), n = 5–6 per group. Adipoq‐F2‐KO mice gained significantly less weight than male counterparts after 12 weeks of diet exposure. Data are presented as mean ± SEM; statistical significance was assessed by two‐way ANOVA (for time courses) or Student's t‐tests (for single time points), with p < 0.05 considered significant. [file PHY2-14-e70875-s005.pdf]

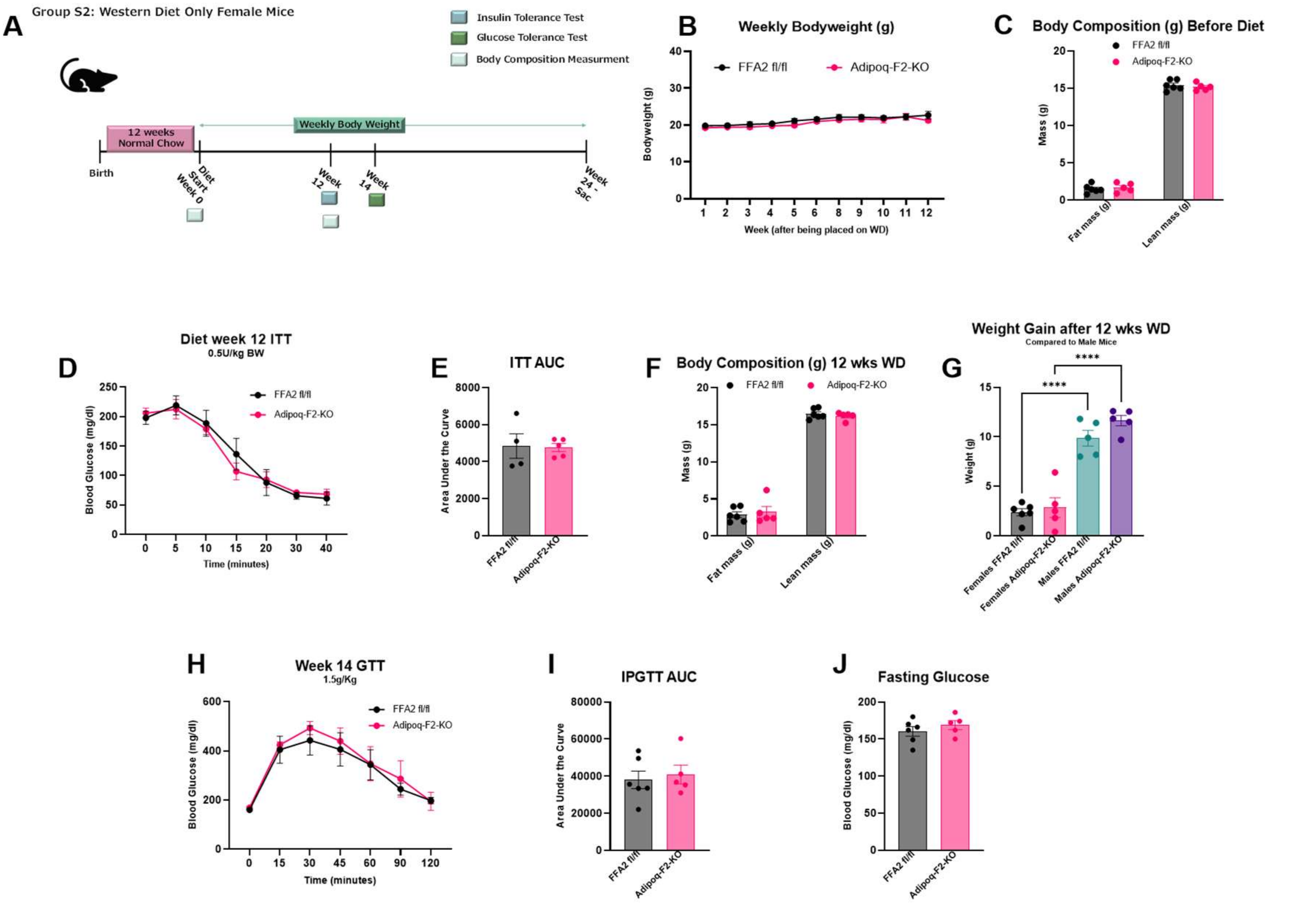

Supplementary Figure 2: Adipoq-F2-KO Female Mice are metabolically comparable to floxed controls on Western Diet.
(A) Schematic of experimental timeline. (B) Weekly body weights measured over 12 weeks of diet treatment showing no difference in weight gain between the two genotypes, n=5-6 per group. Adipoq-F2-KO mice had similar body composition to floxed controls before diet start (C) and they remained comparable after 12 weeks on WD (F), n=5-6 per group. There were also no differences in insulin sensitivity measured by ITT (D, E), glucose tolerance measured by GTT (H, I), and fasting blood glucose (J), n=5-6 per group. Adipoq-F2-KO mice gained significantly less weight than male counterparts after 12 weeks of diet exposure. Data are presented as mean ± SEM; statistical significance was assessed by two-way ANOVA (for time courses) or Student’s t-tests (for single time points), with $p < 0.05$ considered significant.
