# Supplementary material for: Adipocyte‐specific FFA2 deletion leads to increased adipose inflammation and is associated with altered intestinal lipid handling in mice
Source: Physiol Rep. 2026 May 4;14(9):e70875. doi: 10.14814/phy2.70875 (PMC13139770; doi:10.14814/phy2.70875)
Supplement: Supplementary file 3 — Figure S3: Adipoq‐F2‐KO male mice on WD + FOS are comparable to floxed controls in terms of energy expenditure at room temperature. (a) Carbon dioxide produced (b) Oxygen consumed and (c) Locomotor Activity do not differ between Adipoq‐F2‐KO mice and floxed controls as measured by indirect calorimetry gas exchange systems. Apparent gaps between groups are not significant as measured by area under the curve analysis for (d–e) energy expenditure broken down by recording period (whole day, nightly) or (f–h) Respiratory Exchange Ratio broken down by recording period (whole day, daily, nightly). Cumulative Energy Expenditure (i) and Total Distance in cage (j) also show no difference. n = 5–6 per group. Data are presented as mean ± SEM; statistical significance was assessed by two‐way ANOVA (for time courses) or Student's t‐tests (for single time points), with p < 0.05 considered significant. [file PHY2-14-e70875-s008.pdf]

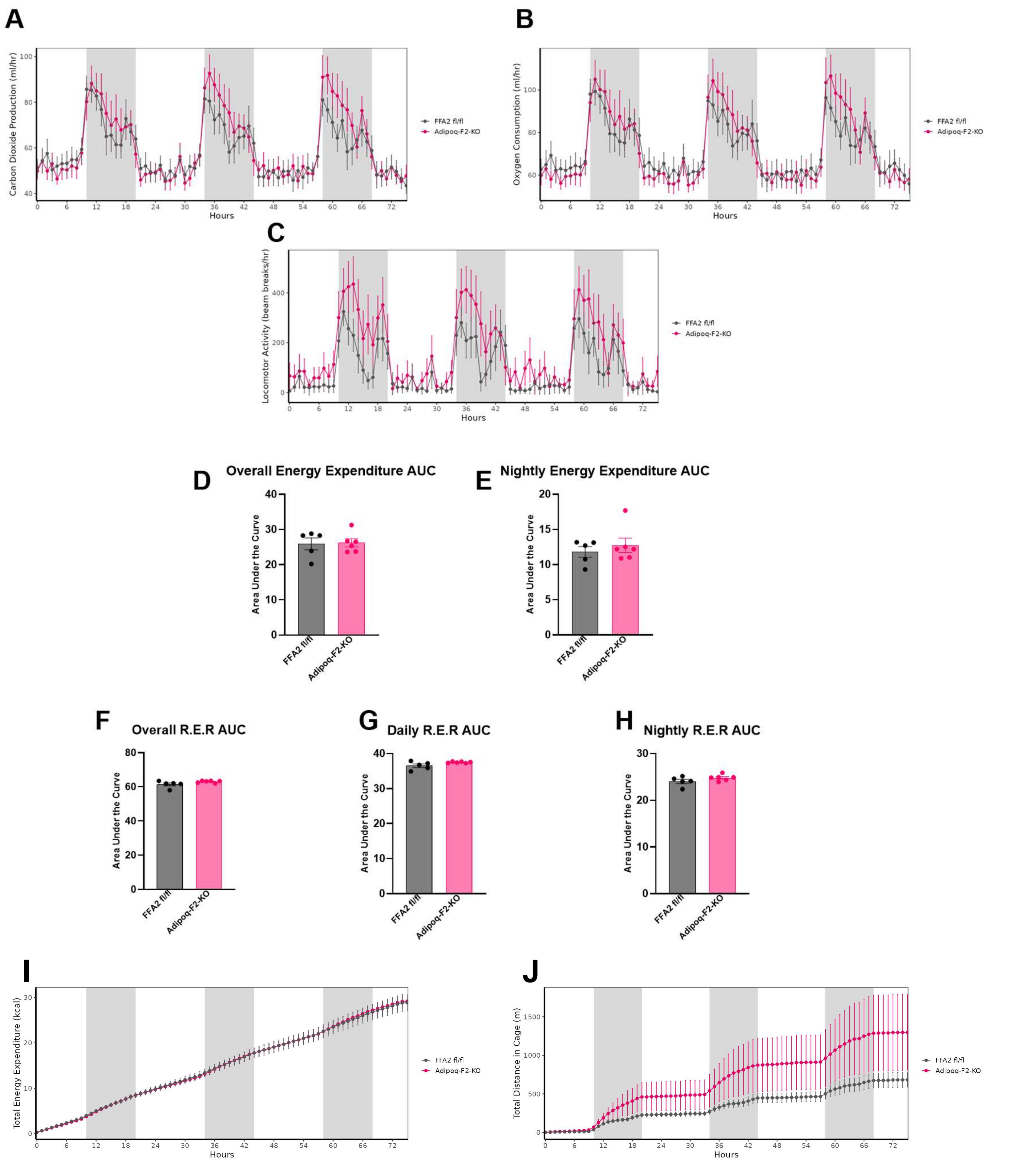


**Supplementary Figure 3: Adipoq-F2-KO Male Mice on WD+FOS are comparable to floxed controls in terms of energy expenditure at Room Temperature.** (A) Carbon dioxide produced (B) Oxygen consumed and (C) Locomotor Activity do not differ between Adipoq-F2-KO mice and floxed controls as measured by indirect calorimetry gas exchange systems. Apparent gaps between groups are not significant as measured by area under the curve analysis for (D-E) energy expenditure broken down by recording period (whole day, nightly) or (F-H) Respiratory Exchange Ratio broken down by recording period (whole day, daily, nightly). Cumulative Energy Expenditure (I) and Total Distance in cage (J) also show no difference. n=5-6 per group. Data are presented as mean ± SEM; statistical significance was assessed by two-way ANOVA (for time courses) or Student's t-tests (for single time points), with $p < 0.05$ considered significant.
