# Supplementary material for: Adipocyte‐specific FFA2 deletion leads to increased adipose inflammation and is associated with altered intestinal lipid handling in mice
Source: Physiol Rep. 2026 May 4;14(9):e70875. doi: 10.14814/phy2.70875 (PMC13139770; doi:10.14814/phy2.70875)
Supplement: Supplementary file 4 — Figure S4: Adipoq‐F2‐KO male mice on WD + FOS are comparable to floxed controls in terms of energy expenditure at thermoneutrality and Adipoq‐F2‐KO mice remain comparable to floxed controls during cold exposure. (a) Carbon dioxide produced (b) Oxygen consumed and (c) Locomotor Activity do not differ between Adipoq‐F2‐KO mice and floxed controls as measured by indirect calorimetry gas exchange systems. (d) Experimental timeline illustrating dietary interventions (WD or WD + FOS) followed by acute cold exposure (4°C) initiated after 6 weeks of dietary challenge. (e) Adipoq‐F2‐KO mice exhibit comparable weight loss to floxed controls following 1 week of cold exposure. (f) Body composition analysis reveals no differences between Adipoq‐F2‐KO and floxed control mice prior to cold exposure. (g, h) Hourly measurements of body weight and core body temperature during cold acclimation. (i) Hourly weight loss during cold acclimation is comparable between groups. (j) Ad libitum blood glucose concentrations remain similar after cold exposure. (k) Body composition measurements after 1 week of cold exposure show no differences between Adipoq‐F2‐KO and floxed controls. Data presented as mean ± SEM; statistical significance assessed by two‐way ANOVA (for time courses) or Student's t‐test (for single time points), with p < 0.05 considered significant. [file PHY2-14-e70875-s001.pdf]

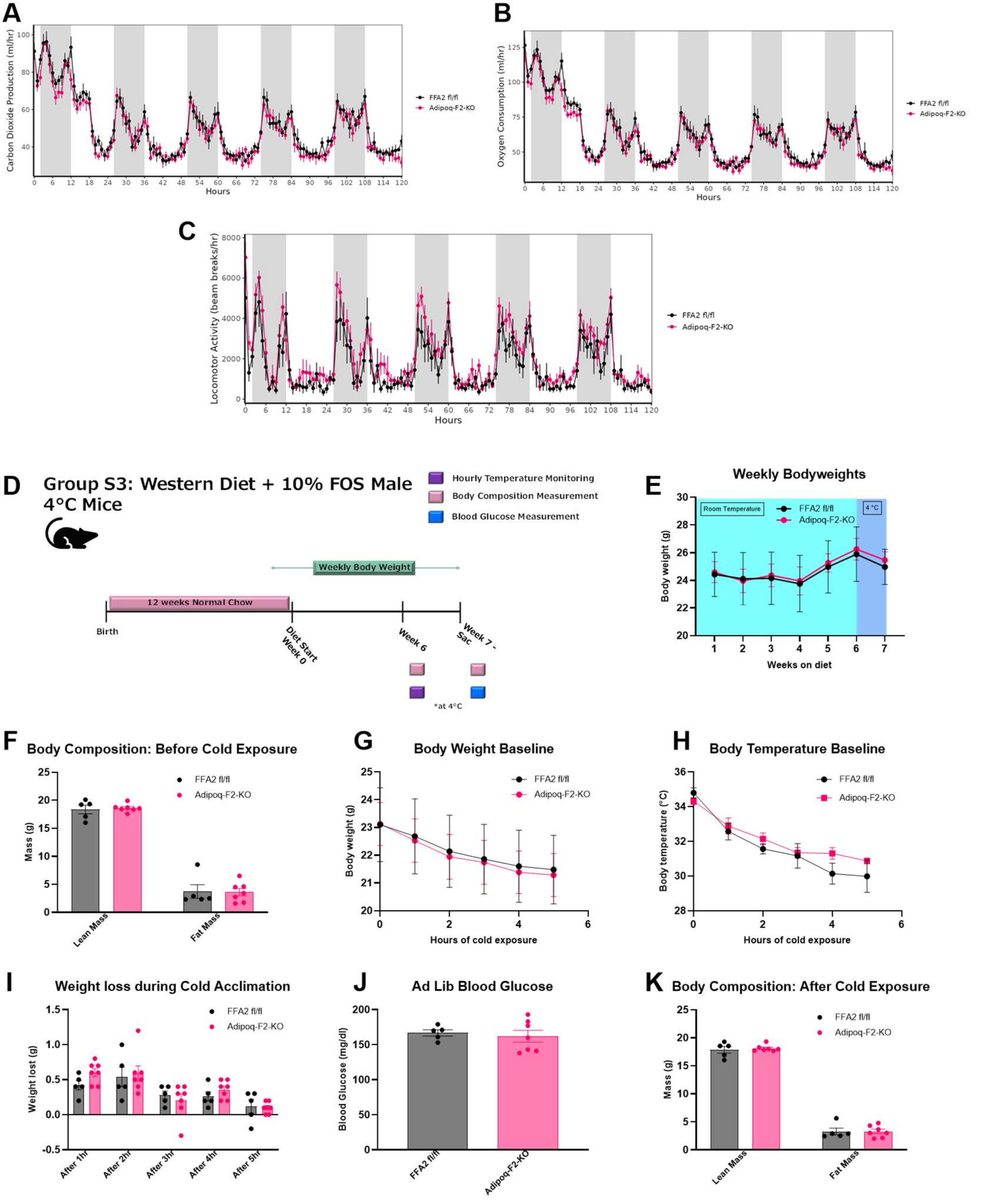

Supplementary Figure 4: Adipoq-F2-KO Male Mice on WD+FOS are comparable to floxed controls in terms of energy expenditure at Thermoneutrality and Adipoq-F2-KO mice remain comparable to floxed controls during cold exposure .
(A) Carbon dioxide produced (B) Oxygen consumed and (C) Locomotor Activity do not differ between Adipoq-F2-KO mice and floxed controls as measured by indirect calorimetry gas exchange systems. (D) Experimental timeline illustrating dietary interventions (WD or WD+FOS) followed by acute cold exposure (4°C) initiated after 6 weeks of dietary challenge. (E) Adipoq-F2-KO mice exhibit comparable weight loss to floxed controls following one week of cold exposure. (F) Body composition analysis reveals no differences between Adipoq-F2-KO and floxed control mice prior to cold exposure. (G,H) Hourly measurements of body weight and core body temperature during cold acclimation. (I) Hourly weight loss during cold acclimation is comparable between groups. (J) Ad libitum blood glucose concentrations remain similar after cold exposure. (K) Body composition measurements after one week of cold exposure show no differences between Adipoq-F2-KO and floxed controls. Data presented as mean ± SEM; statistical significance assessed by two-way ANOVA (for time courses) or Student's t-test (for single time points), with $p < 0.05$ considered significant.
