# Supplementary material for: Adipocyte‐specific FFA2 deletion leads to increased adipose inflammation and is associated with altered intestinal lipid handling in mice
Source: Physiol Rep. 2026 May 4;14(9):e70875. doi: 10.14814/phy2.70875 (PMC13139770; doi:10.14814/phy2.70875)
Supplement: Supplementary file 5 — Figure S5: (a) Oil Red O staining of 3T3L1 EV and F2KD cells after 30 days of differentiation in the presence or absence of 1 mM acetate. (b) Complete Western blot image showing total ERK1/2 detection with molecular weight ladder (Bio‐Rad Precision Plus Protein Dual Color Standards, cat# 1610374). The membrane shown was first probed with anti‐pERK1/2, then stripped and re‐probed with anti‐total ERK1/2 (image shown), and subsequently stripped and re‐probed with anti‐GAPDH (not shown). ERK1/2 proteins migrate at their expected molecular weights of 42/44 kDa, appearing as a doublet between the 37 kDa and 50 kDa ladder markers. GAPDH (37 kDa) migrates at the corresponding ladder position. Molecular weight markers are labeled in kDa. This blot corresponds to Figure 8e in the main manuscript. [file PHY2-14-e70875-s006.pdf]

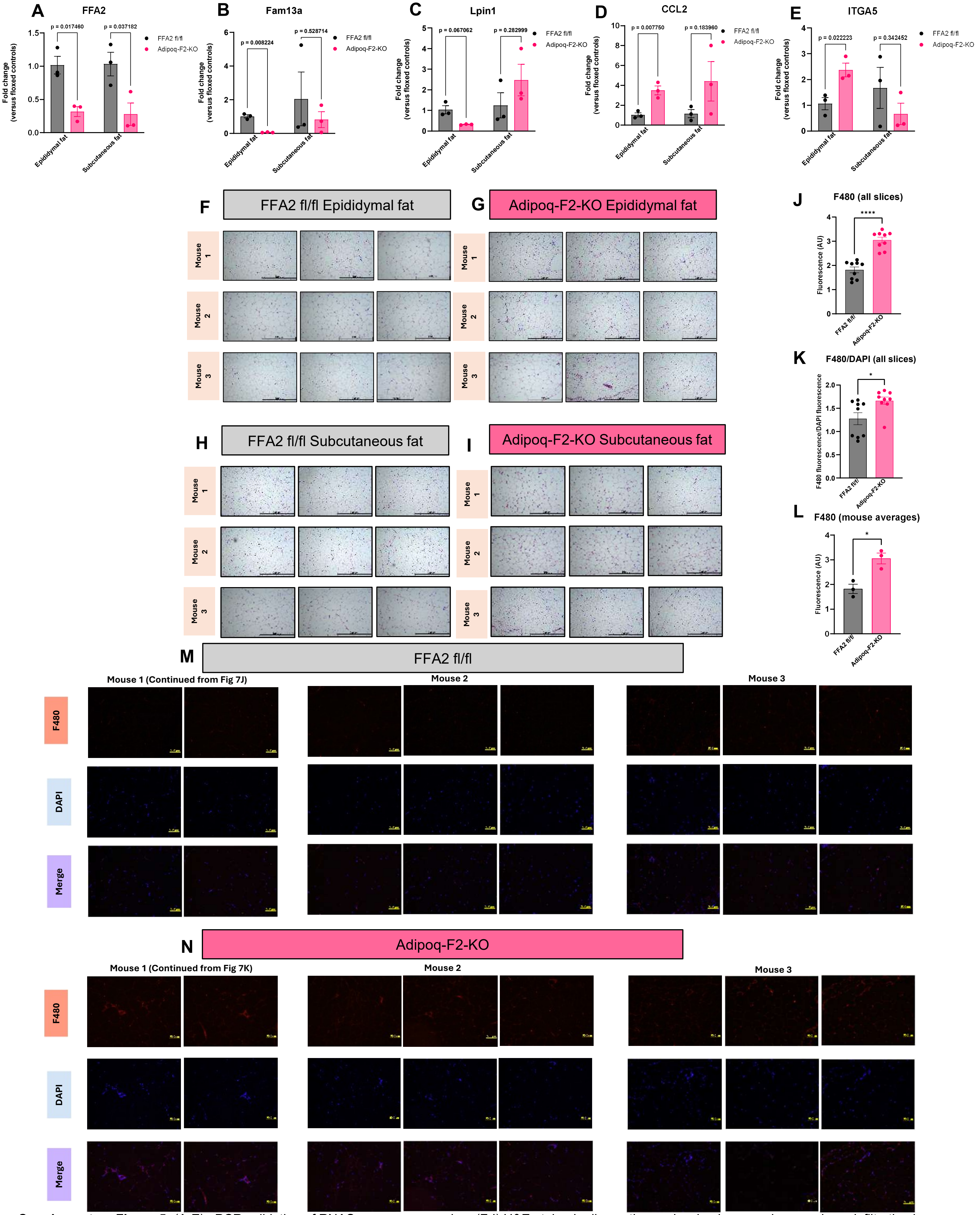

Supplementary Figure 5: (A-E) qPCR validation of RNASeq gene expression. (F-I) H&E-stained adipose tissue showing increased macrophage infiltration in Epididymal fat of Adipoq-F2-KO mice. (J-L) F480 macrophage marker immunofluorescence quantification (M-N) F480 macrophage marker staining confirming increased macrophage presence. N=3 per group. Data presented as mean ± SEM; statistical significance assessed by Student's t-test with $p < 0.05$ considered significant.
