# Supplementary material for: Adipocyte‐specific FFA2 deletion leads to increased adipose inflammation and is associated with altered intestinal lipid handling in mice
Source: Physiol Rep. 2026 May 4;14(9):e70875. doi: 10.14814/phy2.70875 (PMC13139770; doi:10.14814/phy2.70875)
Supplement: Supplementary file 7 — Table S1: Statistical tables showing that Male Adipoq‐F2‐KO gain significantly less weight than floxed controls when given Western diet supplemented with 10% FOS at Room Temperature with the weight divergence being significant from week 12 through week 22 when mice are treated for 24 weeks. [file PHY2-14-e70875-s003.docx]

**Group 3 Weekly Bodyweight Multiple Comparisons**

| **Week** | **Comparison** | **Mean Diff** | **95% CI of diff.** | **Below threshold?** | **Summary** | **Adjusted P Value** |
| --- | --- | --- | --- | --- | --- | --- |
| **Week 1** | Adipoq-F2-KO vs. FFA2 fl/fl | -0.5429 | -2.622 to 1.536 | No | ns | 0.5799 |
| **Week 2** | Adipoq-F2-KO vs. FFA2 fl/fl | -0.8 | -2.780 to 1.180 | No | ns | 0.3938 |
| **Week 3** | Adipoq-F2-KO vs. FFA2 fl/fl | -0.8271 | -3.261 to 1.606 | No | ns | 0.4727 |
| **Week 4** | Adipoq-F2-KO vs. FFA2 fl/fl | -1.429 | -4.035 to 1.178 | No | ns | 0.2522 |
| **Week 5** | Adipoq-F2-KO vs. FFA2 fl/fl | -1.193 | -3.480 to 1.995 | No | ns | 0.43 |
| **Week 6** | Adipoq-F2-KO vs. FFA2 fl/fl | -2.487 | -6.100 to 1.125 | No | ns | 0.1594 |
| **Week 7** | Adipoq-F2-KO vs. FFA2 fl/fl | -1.9 | -5.407 to 1.607 | No | ns | 0.2607 |
| **Week 8** | Adipoq-F2-KO vs. FFA2 fl/fl | -1.992 | -7.161 to 3.178 | No | ns | 0.3692 |
| **Week 9** | Adipoq-F2-KO vs. FFA2 fl/fl | -3.2177 | -7.498 to 1.064 | No | ns | 0.126 |
| **Week 10** | Adipoq-F2-KO vs. FFA2 fl/fl | -2.989 | -7.223 to 1.246 | No | ns | 0.1477 |
| **Week 11** | Adipoq-F2-KO vs. FFA2 fl/fl | -5.4 | -12.36 to 1.563 | No | ns | 0.0901 |
| **Week 12** | Adipoq-F2-KO vs. FFA2 fl/fl | -5.471 | -10.45 to -0.4881 | Yes | * | 0.0346 |
| **Week 13** | Adipoq-F2-KO vs. FFA2 fl/fl | -5.771 | -10.89 to -0.6569 | Yes | * | 0.0308 |
| **Week 14** | Adipoq-F2-KO vs. FFA2 fl/fl | -6.4 | -11.53 to -1.273 | Yes | * | 0.0198 |
| **Week 15** | Adipoq-F2-KO vs. FFA2 fl/fl | -6.843 | -12.47 to -1.216 | Yes | * | 0.022 |
| **Week 16** | Adipoq-F2-KO vs. FFA2 fl/fl | -7.529 | -13.75 to -1.311 | Yes | * | 0.0222 |
| **Week 17** | Adipoq-F2-KO vs. FFA2 fl/fl | -7.943 | -14.51 to -1.380 | Yes | * | 0.0221 |
| **Week 18** | Adipoq-F2-KO vs. FFA2 fl/fl | -7.429 | -13.78 to -1.074 | Yes | * | 0.0259 |
| **Week 19** | Adipoq-F2-KO vs. FFA2 fl/fl | -7.7 | -13.99 to -1.406 | Yes | * | 0.0207 |
| **Week 20** | Adipoq-F2-KO vs. FFA2 fl/fl | -7.014 | -13.16 to -0.8700 | Yes | * | 0.0286 |
| **Week 21** | Adipoq-F2-KO vs. FFA2 fl/fl | -6.386 | -12.47 to -0.3024 | Yes | * | 0.0412 |
| **Week 22** | Adipoq-F2-KO vs. FFA2 fl/fl | -5.486 | -11.86 to 0.8896 | No | ns | 0.0849 |
| **Week 23** | Adipoq-F2-KO vs. FFA2 fl/fl | -4.81 | -15.20 to 5.577 | No | ns | 0.3095 |

**Supplementary Table 1: Statistical tables showing that Male Adipoq-F2-KO gain significantly less weight than floxed controls when given Western diet supplemented with 10% FOS at Room Temperature** with the weight divergence being significant from week 12 through week 22 when mice are treated for 24 weeks.
