# Supplementary material for: Adipocyte‐specific FFA2 deletion leads to increased adipose inflammation and is associated with altered intestinal lipid handling in mice
Source: Physiol Rep. 2026 May 4;14(9):e70875. doi: 10.14814/phy2.70875 (PMC13139770; doi:10.14814/phy2.70875)
Supplement: Supplementary file 8 — Table S2: Adipoq‐F2‐KO Male Mice on WD + FOS are comparable to floxed controls in terms of energy expenditure at Room Temperature (continued). Tables present p values from ANCOVA using total mass (a), lean mass (b), and fat mass (c) as covariates, confirming no effect of mass on energy expenditure parameters as recorded. N = 5–6 per group with p < 0.05 considered significant. Tables present p values from ANOVA of mass‐independent variables (d) over recording timecourse. [file PHY2-14-e70875-s009.docx]

**Statistical Tables (p values) for Group 4 Indirect Calorimetry**

**A. Mass-dependent Variables: ANCOVA - Total Mass as Covariate**

| **Effect** | **Full Day** | **Light** | **Dark** |
| --- | --- | --- | --- |
| Food Consumed (kcal/period) | 0.8930 | 0.3240 | 0.3447 |
| Water Consumed (ml/period) | 0.5233 | 0.2866 | 0.3778 |
| Energy Expenditure (kcal/period) | 0.0608 | 0.3666 | 0.4872 |
| Oxygen Consumption (ml/hr) | 0.5499 | 0.3533 | 0.4060 |
| Carbon Dioxide Production (ml/hr) | 0.0671 | 0.6384 | 0.3742 |

**B. Mass-dependent Variables: ANCOVA - Lean Mass as Covariate**

| **Effect** | **Full Day** | **Light** | **Dark** |
| --- | --- | --- | --- |
| Food Consumed (kcal/period) | 0.3110 | 0.3205 | 0.1983 |
| Water Consumed (ml/period) | 0.3741 | 0.2457 | 0.2597 |
| Energy Expenditure (kcal/period) | 0.2348 | 0.4856 | 0.2609 |
| Oxygen Consumption (ml/hr) | 0.3008 | 0.3648 | 0.2080 |
| Carbon Dioxide Production (ml/hr) | 0.1503 | 0.6444 | 0.7338 |

**C. Mass-dependent Variables: ANCOVA - Fat Mass as Covariate**

| **Effect** | **Full Day** | **Light** | **Dark** |
| --- | --- | --- | --- |
| Food Consumed (kcal/period) | 0.4532 | 0.2912 | 0.2315 |
| Water Consumed (ml/period) | 0.1732 | 0.1403 | 0.2234 |
| Energy Expenditure (kcal/period) | 0.3546 | 0.2000 | 0.5321 |
| Oxygen Consumption (ml/hr) | 0.5642 | 0.3066 | 0.5488 |
| Carbon Dioxide Production (ml/hr) | 0.3516 | 0.3526 | 0.4991 |

**D. Mass-independent Variables (ANOVA)**

| **Effect** | **Full Day** | **Light** | **Dark** |
| --- | --- | --- | --- |
| Pedestrian Locomotion (m) | 0.2962 | 0.2886 | 0.2932 |
| Total Distance in Cage (m) | 0.2841 | 0.2960 | 0.2915 |
| Respiratory Exchange Ratio | 0.9548 | 0.9799 | 0.2209 |
| Locomotor Activity (beam breaks) | 0.1755 | 0.0796 | 0.2387 |

**Supplementary Table 2: Adipoq-F2-KO Male Mice on WD+FOS are comparable to floxed controls in terms of energy expenditure at Room Temperature (continued)**

Tables present p values from ANCOVA using total mass (A), lean mass (B), and fat mass (C) as covariates, confirming no effect of mass on energy expenditure parameters as recorded. N=5-6 per group with p < 0.05 considered significant. Tables present p values from ANOVA of mass-independent variables (D) over recording timecourse.
