# Supplementary material for: Adipocyte‐specific FFA2 deletion leads to increased adipose inflammation and is associated with altered intestinal lipid handling in mice
Source: Physiol Rep. 2026 May 4;14(9):e70875. doi: 10.14814/phy2.70875 (PMC13139770; doi:10.14814/phy2.70875)
Supplement: Supplementary file 9 — Table S3: Adipoq‐F2‐KO Male Mice on WD + FOS are comparable to floxed controls in terms of energy expenditure at Thermoneutrality (continued). Tables present p values from ANCOVA using total mass (a), lean mass (b), and fat mass (c) as covariates, confirming no effect of mass on energy expenditure parameters as recorded. N = 5–6 per group with p < 0.05 considered significant. Tables present p values from ANOVA of mass‐independent variables (d) over recording timecourse. [file PHY2-14-e70875-s007.docx]

**Statistical Tables (p values) for Group 5 Indirect Calorimetry**

**A. Mass-dependent Variables: ANCOVA - Total Mass as Covariate**

| **Effect** | **Full Day** | **Light** | **Dark** |
| --- | --- | --- | --- |
| Food Consumed (kcal/period) | 0.4028 | 0.3866 | 0.4172 |
| Water Consumed (ml/period) | 0.5480 | 0.5734 | 0.4288 |
| Energy Expenditure (kcal/period) | 0.3723 | 0.5068 | 0.2815 |
| Oxygen Consumption (ml/hr) | 0.4055 | 0.2164 | 0.2974 |
| Carbon Dioxide Production (ml/hr) | 0.2499 | 0.1755 | 0.2370 |

**B. Mass-dependent Variables: ANCOVA - Lean Mass as Covariate**

| **Effect** | **Full Day** | **Light** | **Dark** |
| --- | --- | --- | --- |
| Food Consumed (kcal/period) | 0.3650 | 0.2719 | 0.2364 |
| Water Consumed (ml/period) | 0.6680 | 0.4510 | 0.2661 |
| Energy Expenditure (kcal/period) | 0.5913 | 0.5627 | 0.5056 |
| Oxygen Consumption (ml/hr) | 0.5655 | 0.3706 | 0.4145 |
| Carbon Dioxide Production (ml/hr) | 0.8529 | 0.3806 | 0.8725 |

**C. Mass-dependent Variables: ANCOVA - Fat Mass as Covariate**

| **Effect** | **Full Day** | **Light** | **Dark** |
| --- | --- | --- | --- |
| Food Consumed (kcal/period) | 0.6546 | 0.3858 | 0.2670 |
| Water Consumed (ml/period) | 0.1772 | 0.2001 | 0.2495 |
| Energy Expenditure (kcal/period) | 0.3122 | 0.2673 | 0.3062 |
| Oxygen Consumption (ml/hr) | 0.1811 | 0.2162 | 0.2575 |
| Carbon Dioxide Production (ml/hr) | 0.0975 | 0.0995 | 0.1989 |

**D. Mass-independent Variables (ANOVA)**

| **Effect** | **Full Day** | **Light** | **Dark** |
| --- | --- | --- | --- |
| Pedestrian Locomotion (m) | 0.1041 | 1.061 | 1.0011 |
| Total Distance in Cage (m) | 0.1224 | 0.1289 | 0.1185 |
| Respiratory Exchange Ratio | 0.8358 | 0.5939 | 0.5305 |
| Locomotor Activity (beam breaks) | 0.2404 | 0.2981 | 0.2188 |

**Supplementary Table 3: Adipoq-F2-KO Male Mice on WD+FOS are comparable to floxed controls in terms of energy expenditure at Thermoneutrality. (continued)**

Tables present p values from ANCOVA using total mass (A), lean mass (B), and fat mass (C) as covariates, confirming no effect of mass on energy expenditure parameters as recorded. N=5-6 per group with p < 0.05 considered significant. Tables present p values from ANOVA of mass-independent variables (D) over recording timecourse.
